# m6A eraser FTO impairs gemcitabine resistance in pancreatic cancer through influencing NEDD4 mRNA stability by regulating the PTEN/PI3K/AKT pathway

**DOI:** 10.1186/s13046-023-02792-0

**Published:** 2023-08-22

**Authors:** Kai Lin, Endi Zhou, Ting Shi, Siqing Zhang, Jinfan Zhang, Ziruo Zheng, Yuetian Pan, Wentao Gao, Yabin Yu

**Affiliations:** 1 Department of Gastrointestinal Surgery, Affiliated Hangzhou First People’s Hospital, Zhejiang University School of Medicine, Hangzhou, China; 2 Pancreas Center, the First Affiliated Hospital of Nanjing Medical University, Nanjing, China; 3 Department of Hepatobiliary Surgery, The Afliated Huaian No.1 People’s Hospital of Nanjing Medical University, Huaian, China; 4 Medical Faculty of Ludwig Maximilians, University of Munich-Munich, Bayern, Germany

**Keywords:** FTO, N6-methyladenosine, Pancreatic carcinoma, Proliferation, Gemcitabine resistance

## Abstract

**Background:**

Gemcitabine resistance has brought great challenges to the treatment of pancreatic cancer. The N6-methyladenosine (m6A) mutation has been shown to have a significant regulatory role in chemosensitivity; however, it is not apparent whether gemcitabine resistance can be regulated by fat mass and obesity-associated protein (FTO).

**Methods:**

Cells with established gemcitabine resistance and tissues from pancreatic cancer patients were used to evaluate FTO expression. The biological mechanisms of the effects of FTO on gemcitabine resistant cells were investigated using CCK-8, colony formation assay, flow cytometry, and inhibitory concentration 50. Immunoprecipitation/mass spectrometry, MeRIP-seq, RNA sequencing and RIP assays, RNA stability, luciferase reporter, and RNA pull down assays were employed to examine the mechanism of FTO affecting gemcitabine resistant pancreatic cancer cells.

**Results:**

The results revealed that FTO was substantially expressed in cells and tissues that were resistant to gemcitabine. Functionally, the gemcitabine resistance of pancreatic cancer could be enhanced by FTO, while its depletion inhibited the growth of gemcitabine resistant tumor cells in vivo. Immunoprecipitation/mass spectrometry showed that the FTO protein can be bound to USP7 and deubiquitinated by USP7, leading to the upregulation of FTO. At the same time, FTO knockdown significantly decreased the expression level of NEDD4 in an m6A-dependent manner. RNA pull down and RNA immunoprecipitation verified YTHDF2 as the reader of NEDD4, which promoted the chemoresistance of gemcitabine resistant cells. FTO knockdown markedly increased the PTEN expression level in an NEDD4-dependent manner and influenced the chemosensitivity to gemcitabine through the PI3K/AKT pathway in pancreatic cancer cells.

**Conclusion:**

In conclusion, we found that gemcitabine resistance in pancreatic cancer can be influenced by FTO that demethylates NEDD4 RNA in a m6A-dependent manner, which then influences the PTEN expression level and thereby affects the PI3K/AKT pathway. We also identified that the FTO level can be upregulated by USP7.

**Supplementary Information:**

The online version contains supplementary material available at 10.1186/s13046-023-02792-0.

## Introduction

 Pancreatic ductal adenocarcinoma (PDAC) is a highly malignant solid tumor and one of the worst prognosis among digestive tract tumors, with a five-year survival rate of only 12% [1]. The onset of pancreatic cancer is insidious, early diagnosis is difficult, and only 20% of patients have the opportunity to receive radical surgery [2, 3]. Despite the substantial advances in cancer research, PDAC is one of the most chemoresistant malignancies with a high death rate. Therefore, the development of therapeutic strategies to increase the overall treatment efficacy for PDAC patients relies on a deeper understanding of the molecular mechanisms underlying pancreatic cancer chemotherapy, as well as its combination with other therapies to improve the therapeutic outcomes.

Burris et al. first applied gemcitabine to the treatment of pancreatic cancer in 1997 [4]. A number of malignancies can be treated with gemcitabine or 2′,2′-difluoro-2′-deoxycytidine, a nucleoside analogue that interferes with the G1-to-S transition in the cell cycle and induces apoptosis. Gemcitabine works by inhibiting DNA synthesis, repair and maintenance [5–8]. For PDAC, especially those with metastatic lesions, gemcitabine is currently the first-line treatment option [9, 10], while it achieves less favorable results when used to treat pancreatic cancer. The median survival duration for patients with advanced pancreatic cancer receiving gemcitabine alone is approximately 5 months [4]. Gemcitabine and other therapeutic medications have been successful in treating patients with advanced and metastatic PDAC, whereas the development of gemcitabine chemoresistance can significantly restrict treatment efficacy. Both internal and external factors, some of which are specific to this cancer type, contribute to PDAC’s extraordinary capacity to show chemoresistance. Mechanisms that inherently resist gemcitabine can be broadly divided into two categories: (a) mechanisms that hinder drug metabolism until dFdCTP is incorporated into the DNA; and (b) mechanisms that prevent apoptosis after dFdCTP attacks DNA [11]. Various ways can reduce the uptake, metabolism and action of gemcitabine by cancer cells, thereby making them resistant to its toxicity. Regulation of gemcitabine sensitivity by altering molecular pathways involved in nucleoside transport, deoxycytidine kinase, nucleotide reductase, DNA repair, p53, MAPK, NF-κB, histone deacetylase, heat shock protein, fatty acid and sphingolipid metabolism, glycolysis, etc. [11, 12]. Besides, some microRNAs that affect the above molecular pathways can modulate the sensitivity of gemcitabine. microRNAs can control the K-Ras, PI3K-AKT, NF-kB, P53 and Hedgehog pathways in PDAC, which are all connected to gemcitabine resistance in the end [13]. And Environment-mediated gemcitabine resistance (EMDR) is an important factor that contributes to PDAC chemo-resistance and poor prognosis [14]. Tumor microenvironment of PDAC, which consists of extracellular matrix and various non-tumor cells, can also interfere with the delivery and action of gemcitabine, and protect the tumor cells from its toxicity by secreting factors and activating pathways that promote tumor growth, metastasis and survival. Thus, a better clarification of the molecular processes underlying the effect of gemcitabine in the treatment of pancreatic cancer may assist with optimizing the design of improved combination chemotherapies for pancreatic cancer [4, 15].

N6-methyladenosine (m6A), as one of the 170 types of posttranscriptional RNA modifications, most often occurs in mRNAs [16]. Increasing evidence points to the possibility that m6A modification controls RNA processing, splicing, nucleation, translation, and stability, all of which are crucial for the emergence of a variety of human disorders, including cancer [17]. Methylases and demethylases are responsible for the dynamic and reversible process of methylating m6A. A collection of proteins known as "writers" are responsible for transferring methyl groups from the recipient S-adenosylmethionine (SAM) to adenine. These proteins include methyltransferase-like 16 (METTL16), METTL14, METTL3, RNA-binding motif protein 15 (RBM15 and RBM15B), and vir-like m6A methyltransferase-associated protein (VIRMA) (ZCH3H13) [18–20]. IGF2BPs, proteins that bind to the mRNA of insulin-like growth factor 2, as well as proteins from the heterogeneous nuclear ribonucleoprotein (HNRNP) family are examples of binding protein "readers" that may detect m6A methylation [21]. AlkB homolog 5 (ALKBH5) and fat mass and obesity-related protein (FTO) are essential components of "erasers" that enable the reversible process of m6A demethylation [18]. In different phases of many different types of hematomas and solid tumors, the METTL3, FTO and YTH domain families have been linked and may be potential targets for anticancer therapy. The role of m6A and FTO in drug resistance is not well established, but some evidence indicates that they may regulate the expression and stability of genes that affect the chemosensitivity of cancer cells. Recent studies have suggested that m6A modification and FTO expression are involved in the regulation of drug resistance in various cancers, such as acute myeloid leukemia, breast cancer, colorectal cancer, et al. [22–27]. The mechanisms of m6A and FTO-mediated drug resistance may include modulating the expression of drug transporters, DNA repair genes, apoptosis-related genes, and signaling pathways that affect cell survival and proliferation [28]. Therefore, understanding the molecular mechanisms of m6A and FTO in drug resistance may provide new insights for overcoming therapy resistance and improving cancer treatment outcomes. Despite evidence showing that m6A alteration plays a significant role in PDAC, the function and regulatory mechanism of m6A erasers in PDAC development and chemoresistance are still unclear [29–32].

The current work aims to examine the critical regulation of m6A eraser FTO expression and mechanism in PDAC gemcitabine resistance. Our findings indicated that FTO is substantially expressed in gemcitabine-resistant pancreatic cancer tissues. Based on the overexpression and knockdown models, we showed that FTO can improve pancreatic cancer cell resistance to gemcitabine via the FTO-NEDD4-PTEN/PI3K/AKT axis by controlling the cell cycle, thereby influencing cell proliferation.

## Results

### FTO is highly expressed in gemcitabine-resistant pancreatic cancer tissue specimens

**Fig. 1:**
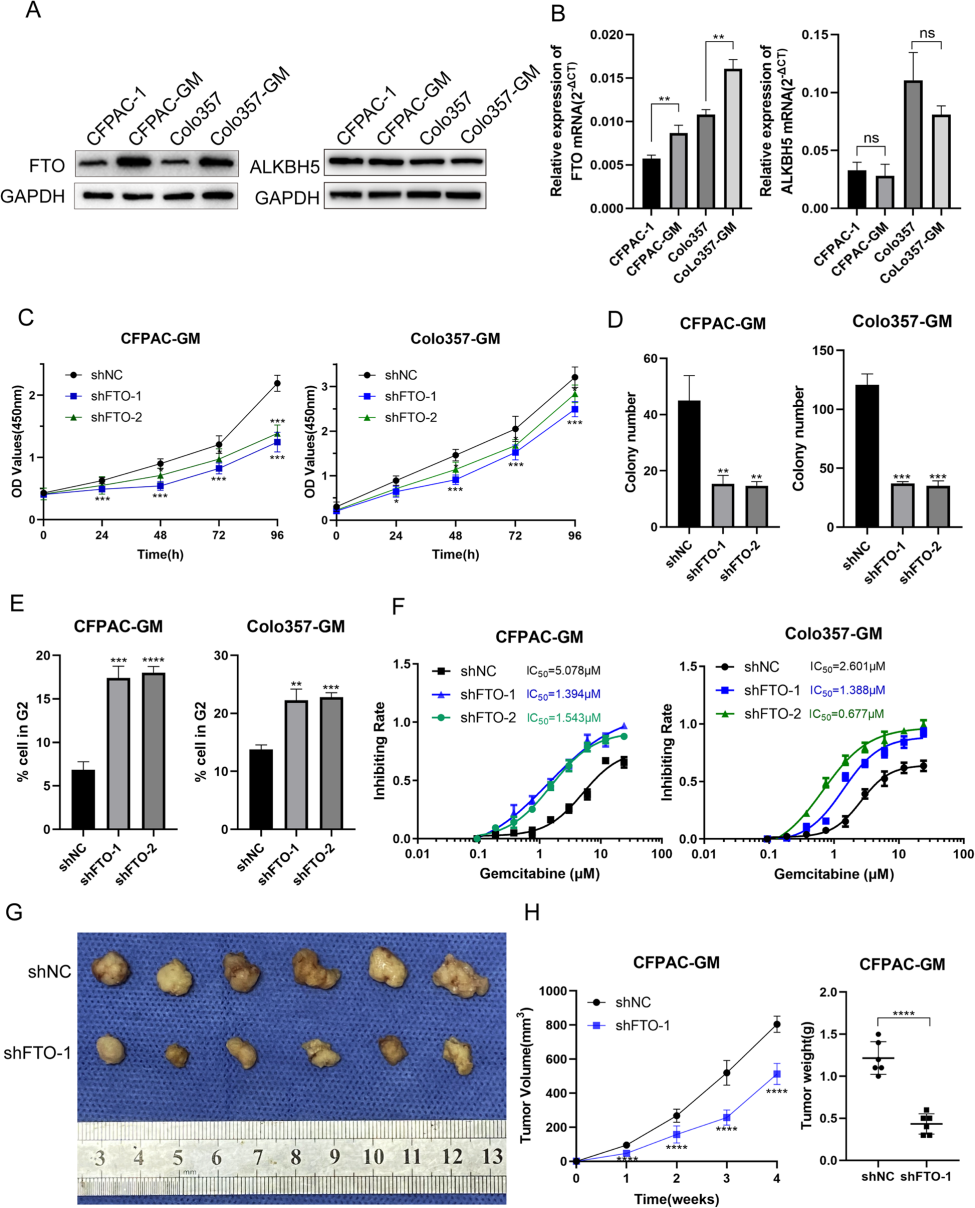
FTO is associated with gemcitabine resistance. **A** FTO and ALKBH5 expression in gemcitabine-resistant and -sensitive cells was evaluated by western blot. **B** Expression level of FTO mRNA in gemcitabine-resistant and -sensitive cells. **C** The findings of the CCK-8 proliferation experiment showed that inhibiting FTO expression reduced the growth of gemcitabine-resistant cells. **D** The ability of CFPAC-GM and Colo357-GM cells to form colonies was inhibited by decreased FTO expression.** E** Cell-cycle profiles of CFPAC-GM and Colo357-GM cells after FTO knockdown. **F** The chemosensitivity of CFPAC-GM and Colo357-GM cells to gemcitabine was assessed using Cell Counting Kit-8 (CCK-8) toxicity tests (GEM). The IC50 values were calculated. **G, H** Silencing FTO in CFPAC-GM cells inhibited pancreatic tumor growth in nude mouse models

We checked the levels of FTO and ALKBH5 expression in gemcitabine-resistant cells to confirm the function of m6A erasers in controlling gemcitabine chemosensitivity in pancreatic cancer. Advanced pancreatic cancer, especially in patients with recurrence and metastasis, is more likely to develop resistance to gemcitabine. Thus, cell lines derived from pancreatic cancer metastases (CFPAC-1 and Colo357) were selected as the research tools for subsequent experiments. We successfully established gemcitabine-resistant cells with CFPAC-1 and Colo357 and named them as CFPAC-GM and Colo357-GM (Fig. S1L). RT-qPCR and western blotting of gemcitabine-resistant cells demonstrated the expression level of FTO and ALKBH5 in these cells (CFPAC-GM and Colo357-GM). Gemcitabine-resistant PDAC cells (CFPAC-GM and Colo357-GM) had considerably greater amounts of FTO mRNA and protein than CFPAC-1 and Colo357 cells (Fig. 1A, B, S9A). However, ALKBH5 remained unchanged (Fig. 1A, B, S9A). Tissues from PDAC patient samples resistant to gemcitabine had a higher expression level of FTO than those sensitive to gemcitabine (Fig. S1A). Collectively, we hypothesized that m6A eraser FTO played a potential role in regulating gemcitabine chemosensitivity in pancreatic cancer. We also verified that FTO mRNAmRNA levels were increased in pancreatic cancer relative to normal tissues in the TCGA and GTEx databases (Fig. S1B), and this was validated in 50 PDAC patient tissues from our hospital (Fig. S1C). And the expression level of FTO is associated with T stage and tumor size (*p* < 0.05, Table 1). In comparison to the normal pancreatic duct cell line HPNE, the FTO mRNA and protein expression levels in the PDAC cell line were relatively high (Fig. S1D, E). Our data from 50 individuals revealed that patients with highly expressed FTO had poor prognosis (Fig. S1F).

**Table 1:** The association between FTO expression and clinical characteristics

Variable	Group	FTO expression		*P* value
		Low (%)	High (%)	
Gender				0.848
	Male	19 (48.7)	20 (51.3)	
	Female	5 (45.5)	6 (54.5)	
Age (year)				0.701
	< 60	5 (38.5)	8 (61.5)	
	≥ 63	20 (44.4)	25 (55.6)	
T				0.024*
	T1 or T2	22 (59.5)	15 (40.5)	
	T3 or T4	3 (23.1)	10 (76.9)	
N				0.765
	0	8 (47.1)	9 (52.9)	
	1 or 2	17 (51.5)	16 (48.5)	
M				0.552
	0	24 (51.1)	23 (48.9)	
	1	1 (33.3)	2 (66.7)	
Stage				0.382
	IA-IIB	14 (45.2)	17 (54.8)	
	III-IV	11 (57.9)	8 (42.1)	
Tumor size				0.020*
	≤ 3 cm	20 (69.0)	9 (31.0)	
	> 3.5 cm	5 (23.8)	16 (76.2)	

### FTO promotes the proliferation and gemcitabine resistance of PDAC cells

In order to show the role of FTO in regulating gemcitabine sensitivity in PDAC, we performed a functional experiment with gemcitabine-resistant cells (CFPAC-GM and Colo357-GM). FTO knockdown and overexpression in gemcitabine-resistant PDAC cells were induced to examine FTO function (CFPAC-GM and Colo357-GM). The CCK8 experiments revealed that FTO knockdown reduced cell proliferation (Fig. 1C), whereas FTO overexpression increased it (Fig. S1G). Colony formation experiments revealed that knocking down FTO reduced the colony formation efficiency (Fig. 1D, S1H), whereas FTO overexpression enhanced colony formation (Fig. S1I, S1J). Furthermore, flow cytometry analysis showed an increase in the proportion of G2 cells in FTO knockdown cells (Fig. 1E, S1K). FTO knockdown lowered the inhibitory concentration 50 (IC50 value) of CFPAC-GM and Colo357-GM cells according to the gemcitabine resistance analysis (Fig. 1F). Additionally, in xenograft models, tumors treated with knockdown FTO cells developed more slowly than those treated with control cells (Fig. 1G, H). The marker of cell proliferation Ki67 also showed reduced expression after FTO knockdown (Fig. S1M). In mice treated with gemcitabine, FTO knockdown in CFPAC-GM cells also reduced tumor growth in vivo (Fig. 5H, I). FB23-2 is an inhibitor of FTO, which can inhibit the demethylase activity of FTO [33]. We used FB23-2, a specific and powerful FTO inhibitor, to examine how FTO inhibition influences cell proliferation and drug resistance. FB23-2 successfully inhibited FTO demethylase activity in PDAC cells (Fig. S2A). Furthermore, we showed that FB23-2 decreased PDAC cell proliferation and increased gemcitabine responsiveness in vitro and in vivo (Fig. S2B-E). Above all, FTO is vital to PDAC cells proliferation and gemcitabine resistance.

### USP7 deubiquitinates FTO proteins

**Fig. 2:**
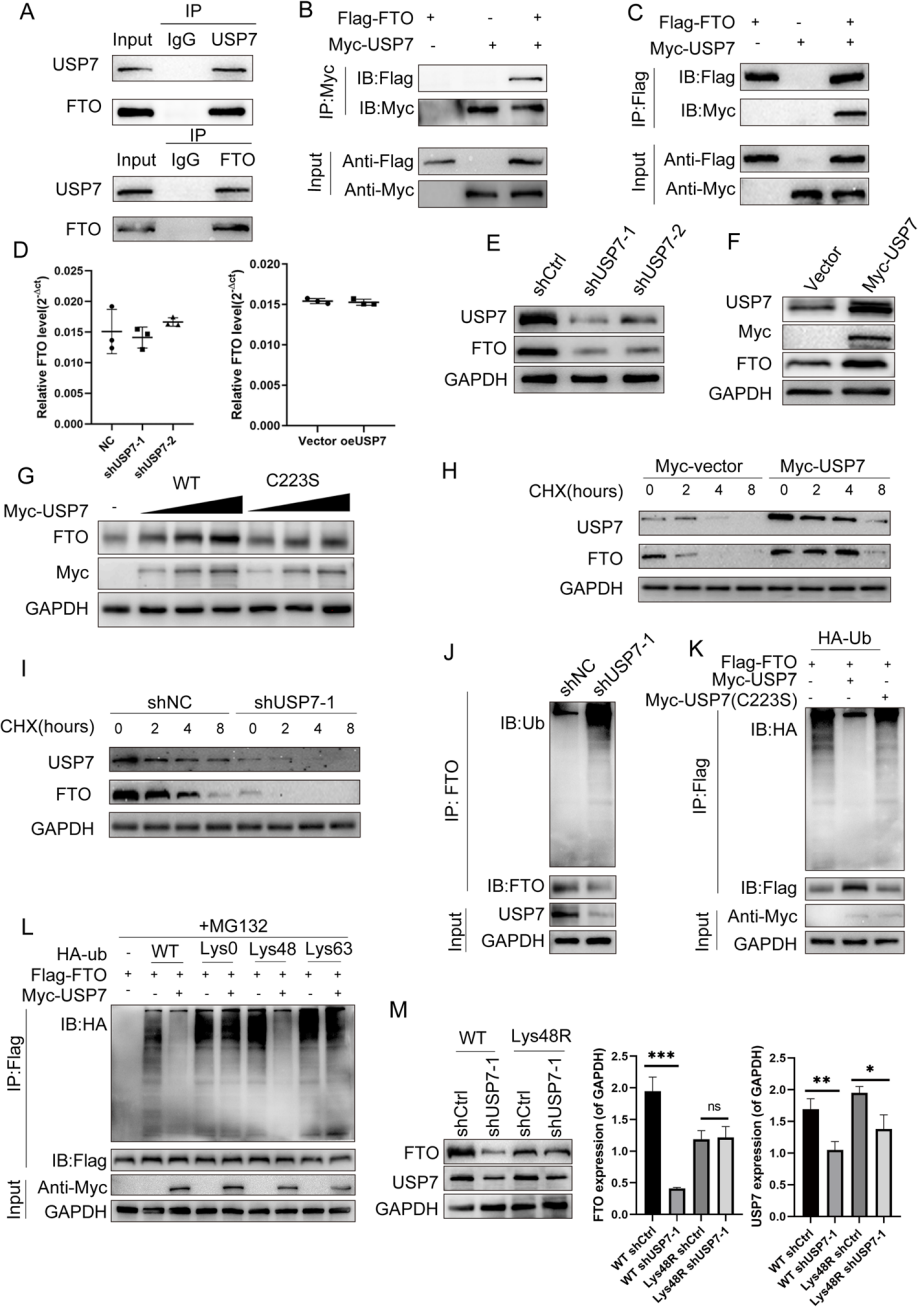
USP7 interacts with and stabilizes FTO **A** Endogenous protein interactions in HEK 293 T lysates were confirmed by immunoprecipitation with anti-USP7 or anti-FTO and immunoblotting with anti-FTO or anti-USP7. **B**,** C** Interactions between exogenous proteins were confirmed in HEK 293 T cells. Lysates from HEK 293 T cells that had been transfected with the USP7 and FTO plasmids were used for an immunoprecipitation reaction with anti-Myc/Flag. **D** FTO mRNA expression in HEK 293 T cells treated with shNC and shUSP7. **E**,** F** Western blotting revealed the levels of FTO and USP7 proteins in HEK 293 T cells transfected with shUSP7 or Myc-USP7 plasmids. **G** Immunoblotting with anti-FTO and anti-Myc was performed to analyze cell lysates after transfection of HEK 293 T with escalating doses of Myc-tagged USP7 (WT or C223S mutant).** H** The FTO protein levels in Myc-Vec and Myc-USP7 HEK 293 T cells were determined by immunoblotting with anti-FTO and anti-USP7 in the presence of cycloheximide (CHX, 10 g/mL) at the designated time points. **I** The FTO expression levels in shNC and shUSP7 HEK 293 T cells were determined by immunoblotting with FTO and USP7 antibody with CHX (10 g/mL) at the designated time points. **J** HEK 293 T cells that had been treated with MG132 and had USP7 knocked down before being harvested resulted in immunoprecipitated lysates that were examined using the relevant antibodies. **K** Lysates from transfected HEK 293 T cells with Myc-tagged USP7 (WT) or Myc-tagged USP29 (C223S), together with HA-tagged Ub and Flag-tagged FTO, were generated after immunoprecipitation with anti-Flag and immunoblotting with anti-HA and anti-Flag. **L** The Myc-USP7, Flag-FTO and the indicated HA-Ub, Lys0, Lys48-only, or Lys63-only plasmids were co-transfected into HEK 293 T cells before the FTO ubiquitylation linkage was assessed. **M** HEK 293 T cells with wild-type Ub or Lys48R mutation were cultured for 72 h in the presence of shNC or shUSP7. Anti-FTO and anti-USP7 immunoblotting was performed to evaluate the cell lysates

In order to investigate the regulation of FTO at the protein level, we performed IP/MS in CFPAC-GM cells. By co-analyzing the IP/MS results and GEO database (GSE80617, ∣log2FC∣ > 1, *P* < 0.05), six proteins (THBS1, CCDC88A, RAN, CCDC138, TRIM47, and USP7) were identified to be upregulated and potentially interacting with FTO in gemcitabine resistant cells. Furthermore, we searched the correlation between FTO and the six proteins on the GEPIA website (http://gepia.cancer-pku.cn/) and found that USP7 had relatively high correlation with FTO (Additional file 12: Supplementary file 1) and was reported to be associated with multi-cancer chemoresistance. Thus, we chose USP7 as a potential regulator of FTO. Ubiquitination is common in proteins and USP7 may be a potential FTO deubiquitinating enzyme (Additional file 13: Supplementary file 2). Reverse Co-IP studies showed that FTO and USP7 may both considerably precipitate each other (Fig. 2A). USP7 and FTO were epitope-tagged when co-IP was performed using HEK 293 T cells, and the results showed that Myc-tagged USP7 co-precipitated with Flag-tagged FTO and Flag-tagged FTO co-precipitated with Myc-tagged USP7 (Fig. 2B, C), demonstrating that USP7 interacted with FTO.

After confirming that USP7 binds to FTO, we studied the influence of USP7 on FTO expression levels. The fact that USP7 overexpression or silencing altered FTO protein levels but had no effect on FTO mRNA levels indicated that USP7 regulates FTO at the protein level but not the mRNA level (Fig. 2D-F, S9B and C). The elevated levels of FTO protein induced by the overexpression of USP7 were unaffected by overexpression of the catalytically inactive C223S variant of USP7 (Fig. 2G, S9D) [34, 35]. In addition, it was examined how the protein synthesis inhibitor cycloheximide (CHX) influenced the stability of endogenous FTO protein when USP7 was either overexpressed or downregulated. USP7 knockdown significantly increased FTO degradation while USP7 overexpression markedly reduced FTO degradation (Fig. 2H, I, S9E, F). We next examined whether the capacity of USP7 to stabilize FTO is related to its function as a deubiquitinase. Compared to shNC, inhibiting USP7 enhanced FTO ubiquitination but decreased FTO protein levels (Fig. 2J, S9G). HEK 293 T cells were transfected with both Myc-USP29 (wild-type or with C223S mutation), HA-Ub, and Flag-FTO to confirm that USP7 controls the ubiquitination of FTO. The findings showed that C223S mutant USP7 transfection had almost no effects on FTO ubiquitination, whereas WT USP7 overexpression reduced it (Fig. 2K, S9H). Polyubiquitin chains come in two different varieties: Lys48-linked and Lys63-linked. The USP7 degraded polyubiquitin chains were linked to lys48 from FTO, whereas Lys63-linked polyubiquitin chains were unaffected (Fig. 2L). To demonstrate that Lys48-linked polyubiquitination is necessary for the USP7 regulation of FTO protein degradation, HEK 293 T cells were transfected with a Lys48-resistant (Lys48R) variant of ubiquitin. The effect of USP7 silencing on the levels of FTO protein was abolished by the overexpression of Lys48R ubiquitin (Fig. 2M). These findings indicated that USP7 modulates the stability of the FTO protein. The same result was observed in PDAC cell CFPAC-GM experiments (Fig. S3A-H, S4A-C).

### NEDD4 is a downstream target of FTO

**Fig. 3:**
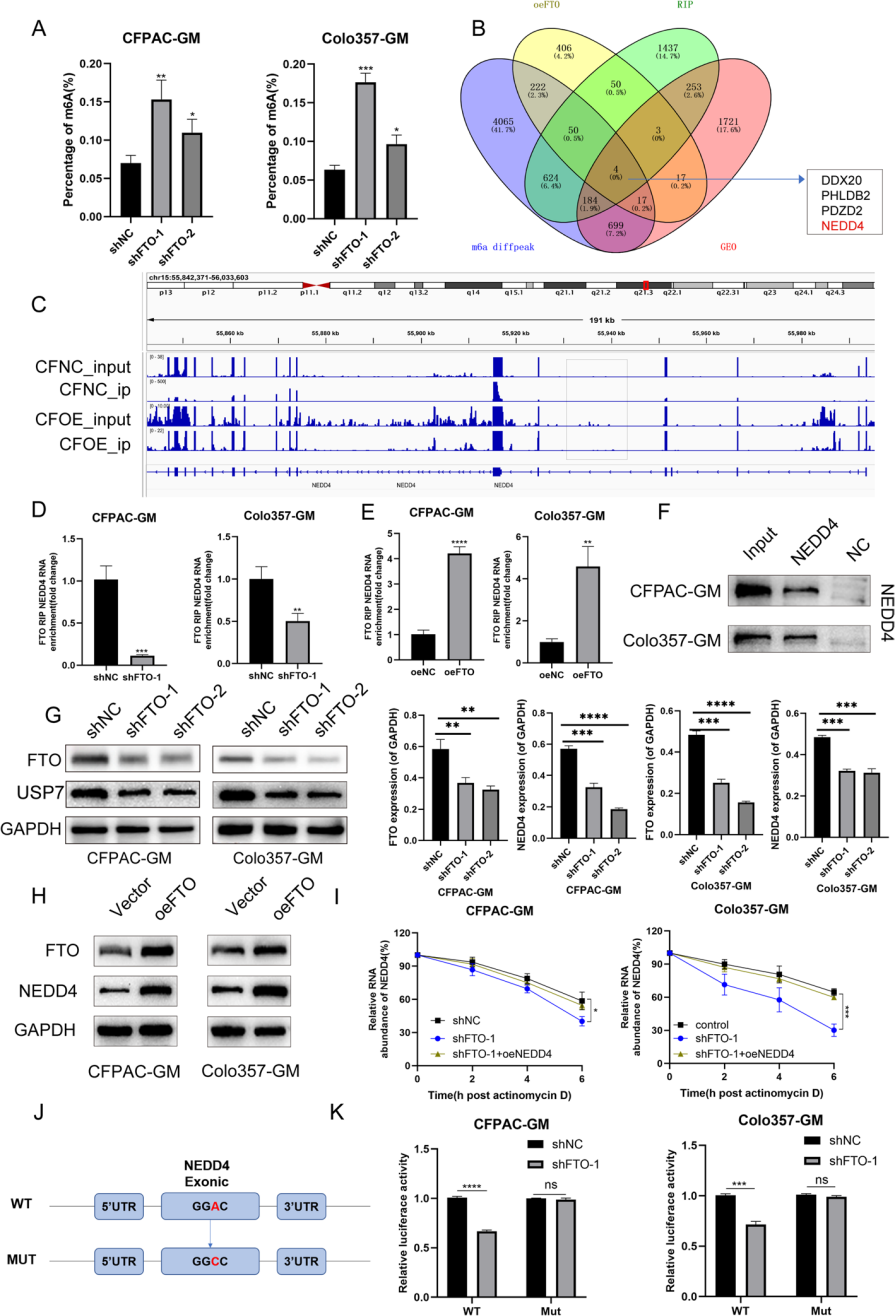
NEDD4 is a downstream target of FTO **A** m6A quantification assay in cells with shFTO. **B** Venn diagram showing a substantial and significant overlap of different peaks of m6A-enriched genes in resistant cells, with the RIP seq of genes bound to FTO, and different genes in FTO-overexpressing cells and database from GEO (GSE80617). **C** Different m6A peaks are enriched in the exonic region of NEDD4, as determined by m6A-seq. **D**,** E** RIP-qPCR assay was conducted to demonstrate the FTO-regulated NEDD4 m6A modification in CFPAC-GM and Colo357-GM cells. **F** FTO antibody was used to identify the results of an RNA pull-down test using NEDD4 full-length RNA. **G**,** H** FTO regulates NEDD4 expression in CFPAC-GM and Colo357-GM cells. **I** RNA stability assay showed that FTO can maintain NEDD4 mRNA stability. **J** NEDD4 cDNA was fused with either the WT or the mutant of m6A consensus sequence firefly luciferase reporter. **K** The relative activity of the WT or Mut luciferase reporters of NEDD4 was measured in FTO-silenced CFPAC-GM and Colo357-GM cells and normalized to the negative control groups

An m6A quantification experiment was performed to confirm the demethylation of m6A by FTO in whole RNA. FTO knockdown significantly enhanced the overall m6A level in PDAC cells (Fig. 3A). To determine the downstream target controlled by FTO, RNA-seq was employed in CFPAC-GM cells with FTO overexpression, that is, MeRIP-seq in CFPAC-GM cells overexpressing FTO and control cells and RIP-seq using FTO antibody. RNA-seq revealed that 769 transcripts were significantly altered by FTO overexpression (∣log2FC∣ > 0, *P* < 0.05) (Additional file 14: Supplementary file 3), and MeRIP-seq revealed 5865 distinct m6A peaks due to FTO overexpression (*P* < 0.05) (Additional file 15: Supplementary file 4). According to the RIP-seq results, 2605 transcripts were bound to the FTO protein (log2FC > 1, *P* < 0.05) (Additional file 16: Supplementary file 5). To uncover gemcitabine resistance-related genes controlled by FTO, the GEO database (GSE80617, ∣log2FC∣ > 0, *P* < 0.05) was co-analyzed. Surprisingly, four transcripts in the sequencing data above were overlapped (Fig. 3B). NEDD4 was chosen as a potential target of FTO-mediated m6A alteration because it was demonstrated to be linked to chemoresistance. As shown in Fig. 3C, FTO overexpression altered the m6A peak of NEDD4. The binding was validated using RIP-qPCR in PDAC cells with FTO knockdown or overexpression. When FTO was overexpressed, NEDD4 enrichment increased, while FTO knockout caused a significant decrease (Fig. 3D, E). The RNA pull-down test revealed that the whole length of NEDD4 mRNAmRNA was bound to the FTO protein (Fig. 3F). FTO knockdown lowered the NEDD4 protein levels whereas FTO overexpression raised them (Fig. 3G, H, S9I). Furthermore, FTO knockdown resulted in decreased mRNA mRNA stability due to the shorter half-life of the NEDD4 transcript following actinomycin D treatment (Fig. 3I). To investigate the impact of m6A alteration on NEDD4 expression, luciferase reporters carrying either WT or NEDD4 mutation were used to investigate the influence of m6A modification on NEDD4 expression. In the mutant version of NEDD4, the adenosine bases in the m6A consensus sequences (RRACH) were switched out for cytosines, and the m6A modification was eliminated (Fig. 3J). At the same time, the luciferase reporter experiment demonstrated that in the absence of FTO, the transcriptional level of wild-type NEDD4, but not the mutant, became substantially reduced (Fig. 3K), indicating that the regulation of NEDD4 is under the control of FTO-related m6A alteration. These findings revealed that FTO interacts directly with NEDD4 and may control the m6A alteration of NEDD4 mRNA.

### YTHDF2 mediates NEDD4 mRNA expression in an m6A-dependent manner

**Fig. 4:**
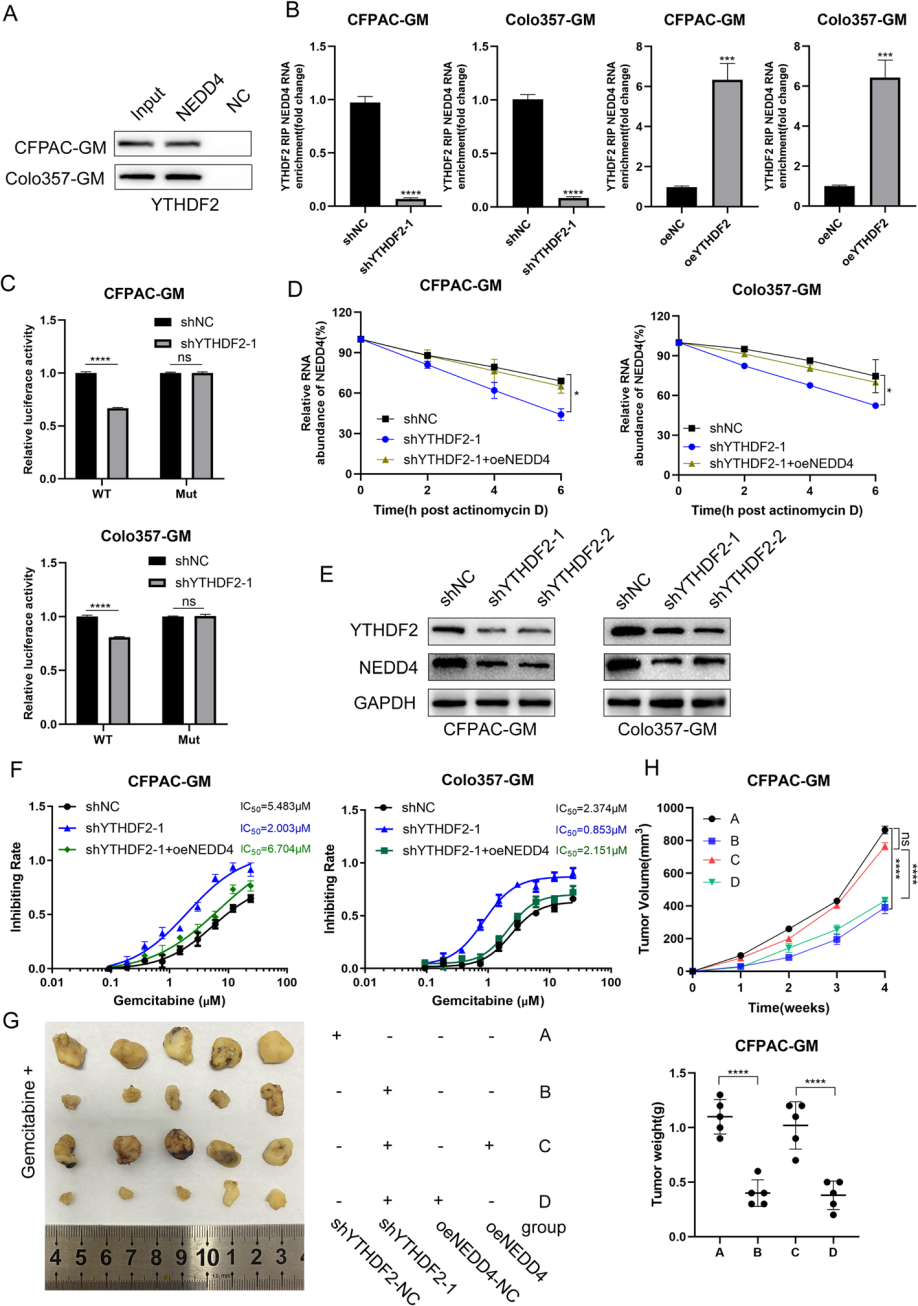
YTHDF2 mediates NEDD4 mRNA expression **A** RNA pull down with NEDD4 RNA detected by YTHDF2 antibody. **B** The NEDD4 m6A alteration caused by YTHDF2 in CFPAC-GM and Colo357-GM cells was shown by RIP-qPCR analysis. **C** In YTHDF2-silenced CFPAC-GM and Colo357-GM cells, the relative activity of the WT or Mut luciferase reporters of NEDD4 was measured and normalized to the negative control groups. **D** RNA stability assay showed that YTHDF2 can maintain NEDD4 mRNA stability. **E** YTHDF2 regulates NEDD4 expression in CFPAC-GM and Colo357-GM cells. **F** The chemosensitivity of CFPAC-GM and Colo357-GM cells to gemcitabine was assessed using CCK-8 toxicity tests. The IC50 of cells transfected with shYTHDF2 and NEDD4 vector were analyzed. **G**,** H** Silencing YTHDF2 or overexpressing NEDD4 in CFPAC-GM cells influenced pancreatic tumor growth in nude mouse models treated with gemcitabine. Group A: cells transfected with shYTHDF2-NC. Group B: cells transfected with shYTHDF2-1. Group C: cells transfected with both shYTHDF2-1 and NEDD4 overexpression plasmid. Group D: cells transfected with shYTHDF2-1 and NEDD4 control vector

The regulatory effects of “m6A readers” on m6A-modified transcripts were recently verified. YTHDFs, including YTHDF1, 2 and 3, are unique m6A readers capable of recognizing and binding to thousands of mRNA transcript targets through the m6A motif and are essential for mRNA stability. Herein, a streptavidin RNA pull-down experiment was carried out to identify the specific m6A readers of NEDD4 and to decipher the m6A-dependent mode of NEDD4 regulation. In PDAC cells, YTHDF2 bound the NEDD4 full-length transcripts (Fig. 4A), and NEDD4 mRNA levels reduced when YTHDF2 was knocked down but increased when YTHDF2 was overexpressed (Fig. 4B). As revealed in Fig. 4C, m6A mutant sites in the NEDD4 mRNA restricted YTHDF2 binding. Furthermore, YTHDF2 deletion decreased the stability of NEDD4 mRNA (Fig. 4D), and YTHDF2 knockdown resulted in a significant decrease in NEDD4 expression while YTHDF2 overexpression exerted the opposite effect (Fig. 4E, S5A, S9J). Gemcitabine resistance studies revealed that knocking down YTHDF2 lowered the IC50 value of CFPAC-GM and Colo357-GM cells (Fig. 4F), which was reversed by the overexpression of NEDD4 in YTHDF2 knockdown cells. In xenograft models treated with gemcitabine, the knockdown of YTHDF2 in CFPAC-GM cells resulted in relatively slow tumor growth. When silencing YTHDF2 and overexpressing NEDD4, the growth rate of the tumor was restored (Fig. 4G, H). Taken together, our findings showed that NEDD4 mRNA stability and expression are controlled by YTHDF2 in an m6A-dependent manner.

We further explored the relevance of FTO, YTHDF2 and NEDD4. Using western blot, we found that FTO overexpression led to an upregulation of NEDD4, and knockdown of YTHDF2 significantly reduced NEDD4 expression. However, overexpressed FTO could not upregulate NEDD4 expression in YTHDF2 knockdown cells. Thus, the results showed that NEDD4 expression was regulated by FTO in a YTHDF2-dependent manner.

### A high level of NEDD4 expression is linked to poor prognosis for PDAC and promotes both in vivo and in vitro chemoresistance in pancreatic cancer

**Fig. 5:**
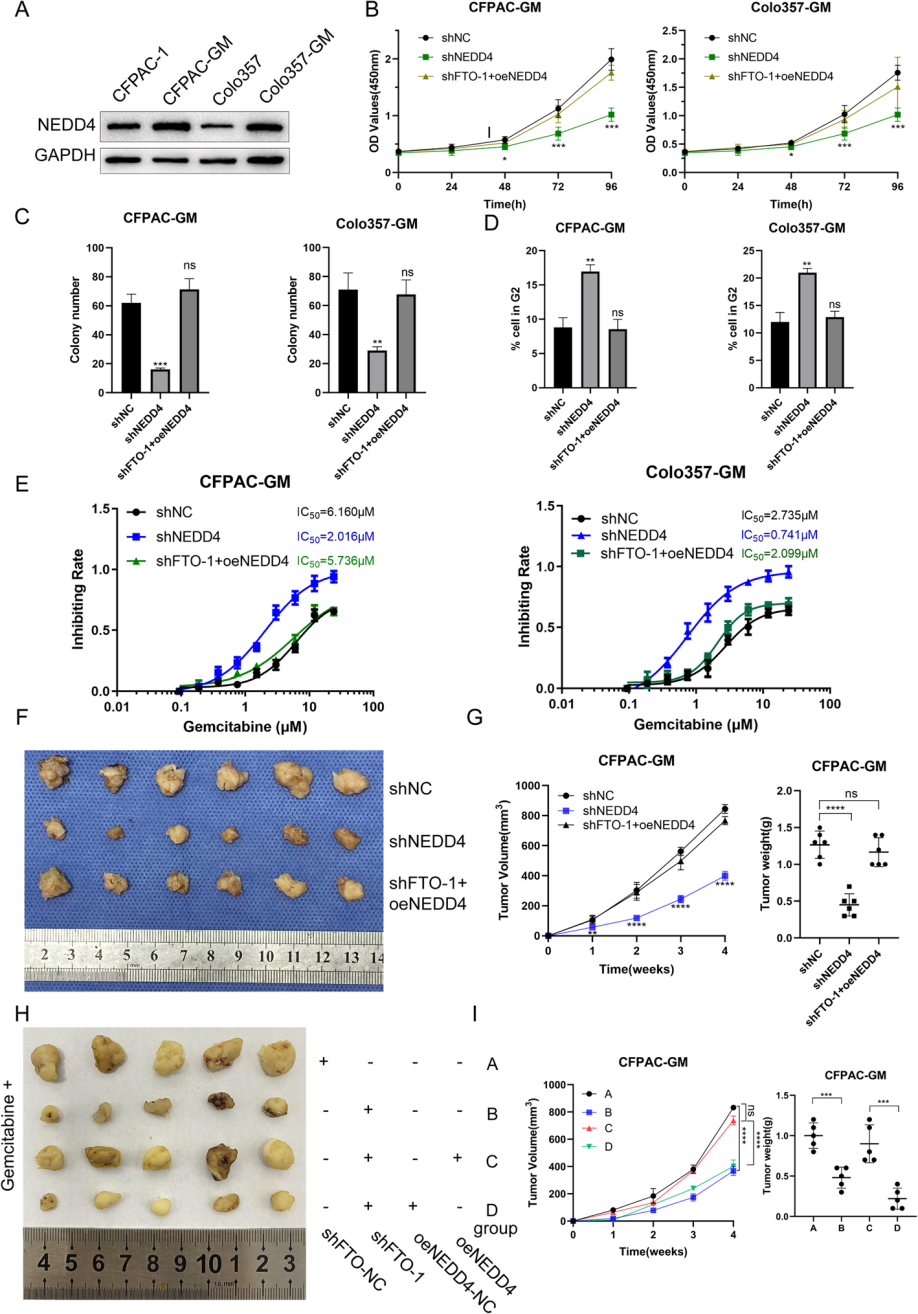
NEDD4 promotes the proliferation of gemcitabine-resistant cells** A** NEDD4 expression in gemcitabine-resistant and -sensitive cells was evaluated by Western blot. **B** CCK8 proliferation assay in cells with shNEDD4 and shFTO with NEDD4 vector. **C** Colony formation assay of NEDD4. **D** Cell-cycle profiles of CFPAC-GM and Colo357-GM cells after the NEDD4 knockdown and rescue experiment. **E** The chemosensitivity of CFPAC-GM and Colo357-GM cells to gemcitabine was assessed using CCK-8 toxicity tests. The IC50 values were calculated. **F**,** G** Silencing NEDD4 in CFPAC-GM cells inhibited pancreatic tumor growth in nude mouse models. The overexpression of NEDD4 rescued the tumor growth rate caused by shFTO. **H**,** I** Silencing FTO or overexpressing NEDD4 in CFPAC-GM cells influenced pancreatic tumor growth in nude mouse models treated with gemcitabine. Group A: cells transfected with shFTO-NC. Group B: cells transfected with shFTO-1. Group C: cells transfected with both shFTO-1 and NEDD4 overexpression plasmid. Group D: cells transfected with shFTO-1 and NEDD4 control vector

NEDD4 mRNA was more abundant in PDAC tumors than normal tissues in the TCGA and GTEx databases (Fig. S5B). NEDD4 mRNA levels were also higher than expected in PDAC tumor samples from patients of our hospital (Fig. S5C). Patients with high NEDD4 expression had a poor prognosis (Fig. S5D). In gemcitabine-resistant cells and tissues, NEDD4 mRNA and protein levels were increased in comparison to gemcitabine-sensitive samples (Figs. 5A and S9K, S5E, F). The CCK8 test was used to validate the function of NEDD4 in PDAC cells to reveal that NEDD4 knockdown resulted in considerably reduced cell proliferation, which may be reversed by overexpressing NEDD4 in FTO knockdown cells (Fig. 5B). The colony formation experiments further showed that NEDD4 knockdown lowered the colony formation efficiency, whereas NEDD4 overexpression reversed this effect in cells transfected with shFTO (Figs. 5C, S5G). Besides, the proportion of G2 cells increased in NEDD4 knockdown cells but was restored in shFTO-transfected cells when NEDD4 was overexpressed (Fig. 5D, S5H). Our gemcitabine resistance assay revealed that NEDD4 knockdown lowered the IC50 value, while the rescue experiment indicated the opposite tendency (Fig. 5E). In xenograft models, tumors injected with NEDD4 knockdown cells grew more slowly than tumors implanted with control cells (shNC), and this difference was reversed when NEDD4 was overexpressed in FTO knockdown cells (Fig. 5F, G). In xenograft models treated with gemcitabine, FTO knockdown in CFPAC-GM resulted in a relatively slow tumor growth. However, co-transfection with shFTO and oeNEDD4 led to recovery (Fig. 5H, I).

### NEDD4 regulated by FTO and YTHDF2 affects the chemosensitivity of gemcitabine-resistant PDAC cells via the PTEN/AKT/PI3K pathway

**Fig. 6:**
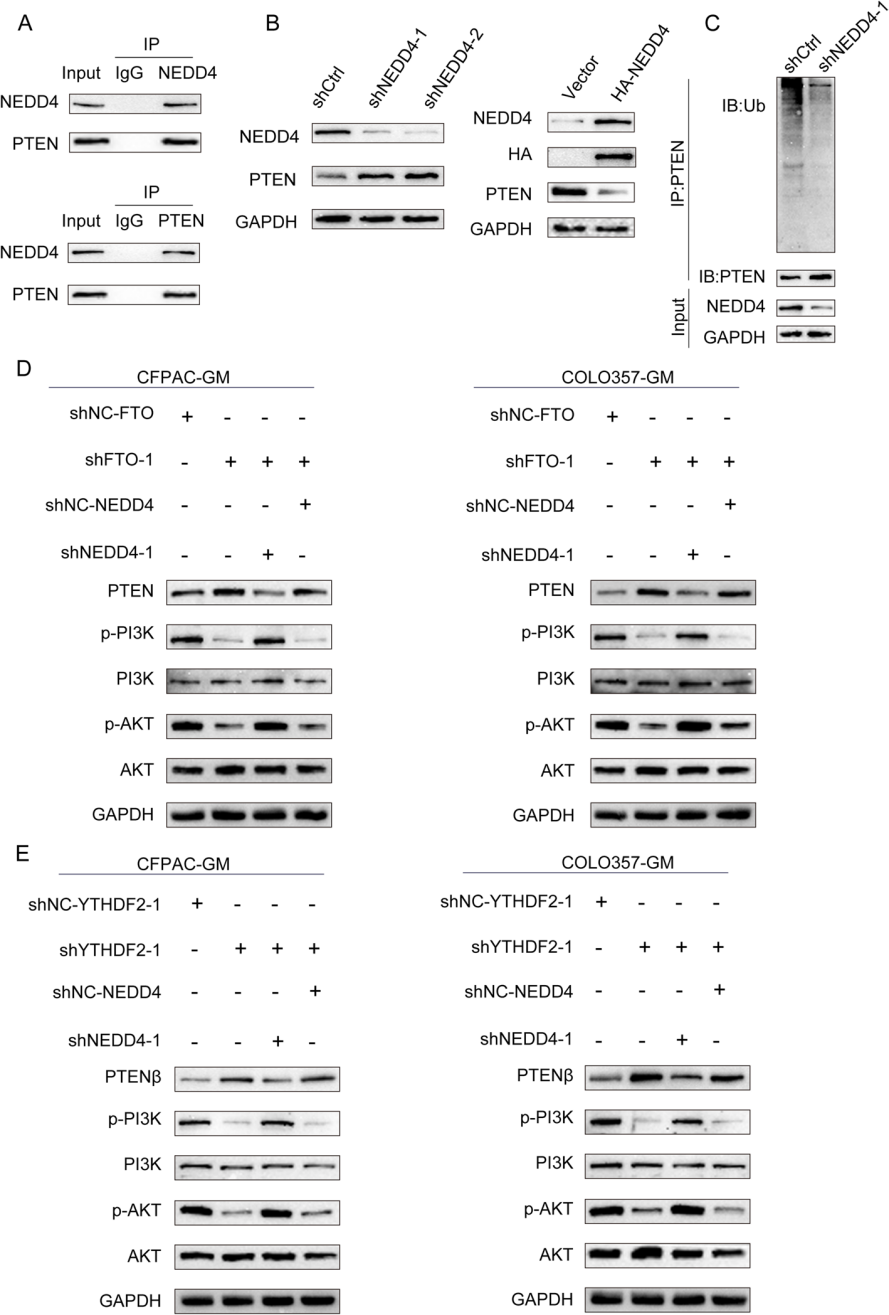
FTO promotes the gemcitabine sensitivity of PDAC cells by regulating the PTEN/AKT/PI3K pathway. **A** Endogenous protein interactions in HEK 293 T lysates were verified by immunoprecipitation with anti-PTEN or anti-NEDD4 followed by immunoblotting with anti-NEDD4 or anti-PTEN, respectively. **B** The levels of the NEDD4 and PTEN proteins in HEK 293 T cells transfected with shNEDD4 or HA-NEDD4 plasmid were analyzed using western blot.** C** After MG132 treatment and shCtrl or shNEDD4 transfection, lysates from HEK 293 T cells were harvested. The cells were then immunoprecipitated and the results were analyzed using the appropriate antibodies. **D**,** E** The protein levels of PTEN, p-PI3K, p-AKT were detected by western blot in CFPAC-GM and Colo357-GM cells with the indicated treatments

**Fig. 7:**
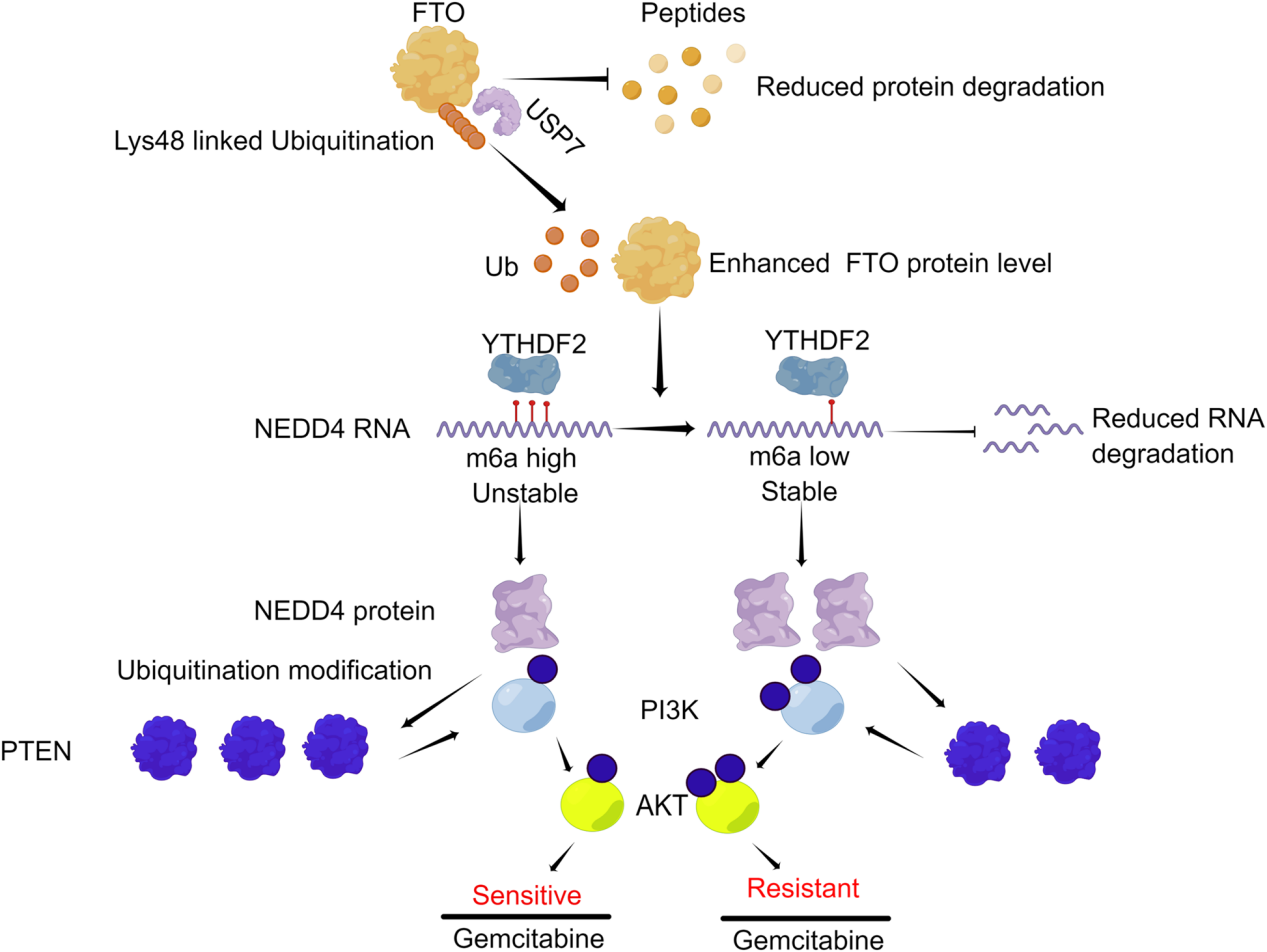
Proposed model: The role and mechanism of FTO in regulating the gemcitabine chemoresistance of PDAC (designed using Figdraw)

NEDD4 is an E3 ligase that can modify protein ubiquitination. To find the NEDD4 modified protein, we searched the STRING and HitPredict databases and found that the tumor suppressor gene PTEN has a high potential to interact with the NEDD4 protein (Additional file 17–18: Supplementary file 6, 7). Then, we conducted an experiment to determine if NEDD4 precipitated PTEN, and the reverse Co-IP results showed that PTEN could also considerably precipitate NEDD4 (Fig. 6A), indicating that NEDD4 interacted with PTEN. NEDD4 silencing or overexpression altered the PTEN protein levels (Fig. 6B, S10A), suggesting that NEDD4 can regulate the PTEN protein level. Subsequently, we identified whether NEDD4 regulates PTEN expression by ubiquitination. Compared to shNC, silencing NEDD4 downregulated PTEN ubiquitination but increased the PTEN protein levels (Fig. 6C, S10B). Meanwhile, the same result can be validated in CFPAC-GM cells (Fig. S4D-F). So far, we can conclude that NEDD4 can regulate PTEN expression in a ubiquitination-dependent manner in PDAC. The PTEN/PI3K/AKT pathway is important in regulating gemcitabine chemosensitivity of pancreatic cancer [36–39]. Therefore, we aimed to investigate whether FTO and NEDD4 affect gemcitabine chemosensitivity by modulating the PTEN/PI3K/AKT pathway. The efficiency of the shRNAs were shown in Fig. S6A and B. When either FTO or NEDD4 was knocked down, significantly more PTEN protein was expressed, but p-PI3K(p85) and p-Akt(s473) were markedly reduced (Fig. 6D, S10C). Conversely, when shFTO and shNEDD4 were co-transfected, the protein expression levels were reversed (Fig. 6D, S10C). FB23-2 was also used to confirm that FTO can regulate the PTEN/PI3K/AKT pathway (Fig. S7A). YTHDF2 was also knocked down, which resulted in higher levels of PTEN protein expression and lower levels of p-PI3K and p-Akt protein expression (Fig. 6E, S11A). Furthermore, the protein levels could be restored by the simultaneous knockdown of YTHDF2 and NEDD4 (Fig. 6E, S11A). And our results also showed that FTO regulates NEDD4 expression in a YTHDF2-dependent manner (Fig. S6C). MK-2206 acts as an allosteric AKT inhibitor. It has high selectivity for all AKT isoforms, namely Akt1, Akt2, and Akt3 [40–43]. In PDAC cells, MK-2206 successfully inhibit AKT activity (Fig. S8A). In the previous experiment, we demonstrated that FTO knockdown can cause G2 phase arrest in pancreatic cancer cells, thereby affecting cell proliferation and regulating their drug resistance. Therefore, we verified that FTO can affect the expression of G2 phase key proteins cyclinB1 and cdc2 by regulating the PI3K/AKT pathway (Fig. S8B). We used MK-2209 in combination with FTO overexpression plasmid and verified by plate clone and I50 in vitro experiments that FTO can indeed regulate the proliferation and resistance of pancreatic cancer cells through the PI3K/AKT pathway (Fig. S8C, D). These results indicate that FTO and NEDD4 are novel regulators of PTEN expression and gemcitabine chemosensitivity in PDAC, and that targeting the FTO/NEDD4/PTEN/PI3K/AKT axis may be a promising strategy to overcome gemcitabine resistance in pancreatic cancer (Fig. 7).

## Discussion

Gemcitabine is a cornerstone drug in the systemic treatment of pancreatic cancer. One of the most frequent genetic alterations in eukaryotic mRNA is m6A methylation, which is implicated in almost all physiological and pathological processes. However, it is unclear if it plays a role in PDAC chemoresistance. Our findings showed that FTO plays an important role in gemcitabine sensitivity in pancreatic cancer cells. We identified the pathway of USP7-mediated deubiquitination modification of FTO, where FTO identified NEDD4 mRNA as a target through m6A alteration. FTO ablation decreased NEDD4 expression and enhanced PDAC cell chemosensitivity to gemcitabine through the PTEN/PI3K/AKT pathway. Furthermore, YTHDF2 was confirmed as an m6A reader of NEDD4 in PDAC cells, and that FTO can regulate NEDD4 RNA stability in a YTHDF2-dependent manner. However, the data on m6A and FTO in drug resistance is still limited and inconsistent, and more studies are needed to elucidate their role in different types of cancers and drugs.

Normal cellular homeostasis is dependent on the balance of protein synthesis and degradation. Intracellular protein degradation is achieved by two systems: the ubiquitin–proteasome system (UPS) and the autophagy-lysosome system. Ubiquitin-specific protease 7 (USP7) is a deubiquitinating enzyme (DUB) that reverses ubiquitination and prevents the degradation of substrate proteins. USP7 has both tumor suppressor and oncogenic roles in cancer development. For instance, non-small cell lung adenocarcinomas have been shown to often present the downregulated expression of USP7 gene, and lower p53 immunostaining was correlated with low USP7 mRNA expression [44]. Given its complex roles in preserving genomic stability, the tumor-suppressive effect of USP7 has been largely attributed to its regulation of p53. However, USP7 also prevents genetic alterations through a number of p53-independent pathways. USP7 may have context-dependent oncogenic effects in addition to tumor suppressive functions [45]. For instance, in a collection of aggressive breast cancers, the levels of the proteins USP7 and geminin were significantly correlated. Poor clinical outcomes have been linked to high geminin protein levels, which were also associated with aneuploidy, errors in DNA replication, and genomic instability. Since geminin is a USP7 substrate, our results imply that USP7 overexpression contributes to breast cancer development by stabilizing geminin [46, 47]. USP7 is an oncogene in pancreatic cancer, and USP7 inhibition blocks PDAC development and promotes cell death in vitro and in vivo [48]. This research also identified the crucial function of USP7 in sustaining chemoresistance [48]; hence, USP7 may be a possible target in tackling chemotherapy resistance.

The fat mass and obesity-related (FTO) gene was the first to be associated with polygenic obesity. In eukaryotic cells, FTO acts as an RNA N6-methyladenosine (m6A) demethylase [49], and the imbalance of m6A has been linked to a variety of human diseases, including obesity, diabetes, heart failure, neurological diseases, and cancer [50–54]. According to the latest research, m6A alterations and their modifying enzymes are dysregulated in a variety of malignancies, including acute myeloid leukemia, glioblastoma, cervical squamous cell carcinoma, breast cancer, and melanoma. Recent research has shown that FTO has an oncogenic impact on pancreatic cancer by directly targeting PDGFC (platelet-derived growth factor C) and stabilizing mRNA expression through m6A [55]. These findings indicate the role of FTO and m6A alterations in carcinogenesis, designating FTO as a possible target for precision therapy in cancer, as discussed further below. Various research groups are now elucidating the molecular involvement of FTO in cancer in terms of its m6A demethylase activity.

NEDD4 is an E3 ligase that takes part in a number of cellular processes by degrading a wide range of substrates through ubiquitination. Cancer development and progression have been linked to the aberrant regulation of the NEDD4 protein. In human malignancies such as gastric, colorectal cancer, NSCLC (non-small-cell lung carcinoma), hepatocellular carcinoma, and others, NEDD4 is commonly overexpressed and is an important factor in medication resistance during cancer treatment. One study found that cisplatin-resistant nasopharyngeal cancer cells overexpress the gene for NEDD4 [56]. Furthermore, NEDD4 knockdown improved cisplatin sensitivity in nasopharyngeal cancer cells that were resistant to the drug, showing that NEDD4 affects the level of sensitivity of cancer cells to it. Similarly, it was shown that erlotinib-resistant NSCLC cells expressed more NEDD4, and NEDD4 promoted erlotinib resistance in NSCLC cells by targeting PTEN degradation [57]. The findings of this study indicated that NEDD4 has the potential to significantly influence tumor treatment resistance.

Gemcitabine (dFdC, 2’, 2’-difluoro- 2’-deoxycytidine) is a nucleoside analog that inhibits DNA synthesis and induces cytotoxicity in various cancer cells1. Gemcitabine is the mainstay of treatment for pancreatic ductal adenocarcinoma (PDAC), a highly lethal malignancy with limited therapeutic options [58]. The PI3K/AKT/mTOR pathway is a key regulator of cellular metabolism, proliferation, angiogenesis, and invasion in response to diverse stimuli such as metabolites, growth factors, and hypoxia [59]. PI3Ks are a class of lipid kinases that have three different types of structure and function. Among them, type I PI3Ks can transform PIP2 into PIP3. In addition, type II PI3Ks can transform PI into PIP, while type III PI3Ks can transform PIP into PIP2 [60–62]. Class I PI3Ks, which have two subtypes, IA and IB, consist of a catalytic subunit of 110 kDa (p110) and a regulatory subunit of 85 kDa (p85). Class IA and IB PI3Ks are triggered by RTKs and GPCRs, respectively, and both transform PIP2 into PIP3 when activated. Poor overall survival in PDAC patients is linked to higher activation of the PI3K signaling [63, 64]. Akt, a serine-threonine kinase from the AGC kinase family, is another vital molecule in the PI3K signaling node. It is brought to the plasma membrane by the interaction of the pleckstrin homology domain and membrane lipids when PI3K receives the activating stimulus. Akt then controls cell growth, proliferation, and survival by phosphorylating various downstream proteins and transcription factors that are related to anti-apoptosis, chemoresistance and cell cycle [36, 65]. And abnormal phosphorylation activation of PI3K/AKT pathway leads to dysfunction of tumor cells [66–70]. Previous studies have shown that inhibitors of PI3K and AKT can modulate gemcitabine sensitivity and induce apoptotic cell death in pancreatic cancer cells [71]. PTEN (phosphatase and tensin homolog) is a tumor suppressor that antagonizes this pathway by dephosphorylating PIP3 and preventing AKT activation [71]. In this study, we found that E3 ligase NEDD4 ubiquitinates PTEN and promotes its degradation, leading to PI3K/AKT pathway activation and gemcitabine resistance.

However, this research has several limitations. The processes underlying the control of FTO mRNA elevation remain unexplored. In addition, the sample size of PDAC patient tissues is relatively small and may not represent the general population..

## Conclusions

Our results showed that FTO is increased in gemcitabine-resistant PDAC cells, indicating a poor prognosis. USP7 may deubiquitinate FTO, resulting in the increased expression of FTO protein, which induces gemcitabine resistance in PDAC cells and enhances NEDD4 mRNA stability in a YTHDF2-dependent manner. FTO knockdown markedly increased the PTEN expression level by regulating NEDD4 expression and influenced the PI3K/AKT pathway, which led to chemoresistance to gemcitabine in pancreatic cancer cells. Thus, targeting FTO may provide potential therapeutic treatment strategies for PDAC patients.

## Materials and methods

### Clinical tissue specimens

PDAC adenoma and adjacent tissues were taken during surgery and received from Jiangsu Province People's Hospital, which is affiliated with Nanjing Medical University. The pathological data corroborated the diagnosis of PDAC and adenoma. Conventionally, the tissues are divided into two parts, one frozen in liquid nitrogen and the other immersed in formalin fixative for tissue embedding. The patient samples included 50 cases of pancreatic cancer patients who had not received neoadjuvant chemotherapy, and we selected 6 patients based on their responsiveness to gemcitabine neoadjuvant chemotherapy (Resistant: Progressive disease, PD, *n* = 3; Sensitive: Complete response, CR, Partial response, PR, Stable disease, SD, *n* = 3) and retrieved their tissue specimens from the sample bank.

### Cell culture, transfection and drug treatment

Plastic or glass cell culture dishes were used to plate PDAC cell lines in DMEM with 10% FBS and penicillin/streptomycin (100 U/mL) in a 5% CO_2_ incubator at 37 °C (NEST Biotechnology). HPNE, Miapaca-2, Panc-1 and CFPAC-1 cells were kindly provided by Cell Bank/Stem Cell Bank, Chinese Academy of Sciences. Colo357 cells were obtained from Heidelberg University where the cells were developed previously [72], and one copy was kept by the University. The overexpression vector, negative control, and short hairpin RNA plasmid (Additional file 19: Supplementary Table S1) were all transfected using the Lip3000 reagent (Invitrogen, USA). And the transfected efficiency would be validated on day 2 after transfection. FB23-2 (10 μM, MCE), MK-2206 (1 μM, Selleck Chemicals) or vehicle control (DMSO) were administrated to PDAC cells and keeping treatment until detection.

### Establishment of gemcitabine-resistant cell lines

Gemcitabine-resistant cells were established by our laboratory. Briefly, CFPAC-1 and Colo357 cells were initially exposed to 0.2 μM of gemcitabine for 1 week. When cells returned to their normal growth rate after the recovery period, the concentrations of gemcitabine were gradually increased (5, 10, 20 and 40 μM) until cells became resistant to 40 μM of gemcitabine, at which point they were designated as CFPAC-GM and Colo357-GM, respectively.

### Quantitative real-time polymerase chain reaction

The NucleoZOL reagent was utilized to extract total RNA (Macherey–Nagel, Germany). The HiScript III RT SuperMix was used to create cDNA for qPCR (Vazyme, China). The ChamQ SYBR qPCR Master Mix (High ROX Premixed) was used to conduct the amplification reaction (Vazyme, China). GAPDH or 18 s were used to normalize gene expression. The primers are listed in the Additional file 20: Supplementary Table S2.

### Western blotting

The protein concentration was evaluated after collecting and lysing cells. The protein was separated, transferred to a PVDF membrane, and then treated with QuickBlock™ Blocking Buffer for western blotting for 15 min at room temperature (Beyotime, China). Primary antibodies were applied to the membrane and left overnight at 4℃. Afterwards, a secondary antibody was used to treat the membranes for an hour at room temperature (1:10,000, Jackson ImmunoResearch). A chemiluminescence gel imaging device was employed to observe protein expression (Thermo Fisher Scientific, FL1500, USA). Antibodies for western blotting were listed in Additional file 21: supplementary Table S3. For PI3K/AKT pathway validation, p-ATK (Ser473) and p-PI3K (p85) site antibodies were used.

### Immunoprecipitation (IP) and IP with mass spectrometry (IP/MS)

Total proteins from CFPAC-GM cells were extracted and then removed after incubation overnight at 4℃ with the appropriate primary antibodies. The next day, protein G from Dynabeads was added and incubated at room temperature for an hour. SDS-PAGE gels were used then with the immunoprecipitated protein (experimental group and IgG group). And proteins were extracted from the full lane of gels. Mass spectrometry was used to analyze the extracted immunoprecipitants. The IP/MS results were analyzed according to immunoprecipitants from the experimental group and IgG group.

### Measurement of m6A modification

The EpiQuik m6A RNA Methylation Quantification Kit (Colorimetric) was used to measure m6A levels in whole RNA (P-9005–48). Briefly, 200 ng of RNA was exposed to the detection antibody and enhancer solution after being treated with capture antibody for 1 h. The samples were then placed in developer solution and incubated for 10 min. The absorbance was determined at a wavelength of 450 nm.

### Immunohistochemistry (IHC)

In paraffin-embedded tissues, IHC was performed to assess protein expression. This was performed using the SP Kit (Broad Spectrum, SP0041, Solarbio). Tissue slides were treated with xylene and 100% ethanol before the ethanol concentration started to decrease. After antigen retrieval, slides were blocked and stained with primary antibodies, and then the secondary antibodies were incubated using the standard avidin biotinylated peroxidase complex method. Hematoxylin acted as a counterstain and an upright microscope was used to acquire the images (Nikon, JAPAN). Immunohistochemistry average optical density (AOD) is a quantitative analysis method to evaluate the expression level of certain peptides and proteins in tissue sections or cell specimens. Integrated optical density (IOD) is the sum of optical density values at each point within the positive expression area. ImageJ was used to for measuring. We calculated AOD by dividing IOD by the area of positive expression. The antibodies we used were listed in Additional file 21: supplementary Table S3.

### Cell proliferation assays

In 96-well plates, 6 × 10^3^ PDAC cells were seeded for the Cell Counting Kit-8 (CCK8) test. CCK-8 (Vazyme), which was added after cell adhesion, was then incubated for an hour at 37℃ and 5% CO_2_ to assess cell proliferation at 0, 24, 48, 72, and 96 h. A microplate reader was used to measure absorbance at 450 nm (TECAN). A total of 1 × 10^3^ cells were plated and cultivated for two weeks on 6-cm^2^ cell plates for the clonogenic test. The cells were stained with 0.1% crystal violet after being fixed with 4% paraformaldehyde (Sigma, St. Louis, MO, USA). Colonies were counted until they could be seen under a light microscope.

### Flow cytometry analysis

After staining with propidium iodide (PI) for cell cycle analysis using a cell cycle kit, the cells were counted with a FACSCalibur flow cytometer in accordance with the manufacturer's instructions (Multisciences, China).

### RNA stability assay

CFPAC-GM and Colo357-GM cells were planted in 6-well plates for 24 h before being treated with 5 μg/mL actinomycin D (MCE, USA) at 0, 2, 4, and 6 h. NucleoZOL was used to extract the total RNA, and qPCR was implemented to examine the results. At each time point, the amount of mRNA in each group was measured and normalized using GAPDH.

### Methylated RNA immunoprecipitation sequencing (MeRIP-seq) and MeRIP-qPCR

The MeRIP-seq was completed in line with a previously published procedure with minor changes by LC-Bio Technology CO., Ltd., Hangzhou, China [73]. To identify particular alterations in the M6A gene, the MeRIP-qPCR method was employed. In conclusion, poly(A) RNA was obtained from 50 ng of total RNA using the Dynabeads mRNA Purification Kit (61,006, Invitrogen), and one-tenth of the RNA was retained as the input control. Prewashed Pierce™ Protein A/G Magnetic Beads (88,803, Thermo Scientific) were incubated with 5 g of either rabbit IgG or anti-m6A antibody for 2 h at 4℃ while being rotated. After three times of washing, pure poly(A) RNA and an immunoprecipitation solution containing RNase inhibitors were mixed with the antibody-attached beads. This was followed by proteinase K digestion, then the methylated mRNAs were precipitated using 5 mg of glycogen and 0.1 volume of 3 M sodium acetate in a 2.5 volume of 100% ethanol overnight at 80℃. qPCR was used to quantify the additional enrichment, and after normalizing each sample to the input, the corresponding m6A enrichment was calculated.

### RNA pull-down assays

The MEGAscript T7 Transcription Kit was used first to transcribe the RNA (Thermo Scientific). Subsequently, the ends of the amplified RNA were desthiobiotin-labeled using the Pierce RNA 3′ End Desthiobiotinylation Kit (20, 163, Thermo Scientific). Last but not least, RNA pull-down experiments utilizing the Pierce Magnetic RNA–Protein Pull-Down Kit were performed (20, 164, Thermo Scientific). Specifically, 2 mg of protein lysates, 50 pmol of biotinylated RNAs, and 50 µL of streptavidin beads were mixed. The streptavidin beads were boiled in preparation for the immunoblotting experiment after three washing cycles and an incubation period.

### Luciferase reporter assay

Luciferase reporters were developed by Genechem using the wild-type (WT) NEDD4 exonic region and mutated-type (Mut) sequences (Chr15: 55,924,457–55,951,517) (Shanghai, China). The WT and Mut sequences of the FTO/YTHDF2 promoter were used by Tsingke to construct luciferase reporters (Nanjing, China). Cells were seeded and transfected with the WT/Mut reporters on a 24-well dish. According to the manufacturer's recommendations, the luciferase test was carried out using the Luciferase Assay System from Promega. A BERTHOL (Centro XS LB 960) chemiluminescence measuring instrument was employed to measure luciferase activity.

### Animal studies

The animal testing procedures followed the institutional ethical norms for animal experimentation set by Nanjing Medical University's animal management committee. Female athymic BALB/C nude mice received a subcutaneous injection of 5 × 10^6^ cells into the axilla (4 weeks old, 18–20 g). The tumor volume (V) was calculated each week using the formula V = (W2 L)/2 after measuring the tumor width (W) and length (L). After the injection of cancer cells, for the gemcitabine treatment cohort, mice were injected with gemcitabine (120 mg/kg, Sigma-Aldrich) intraperitoneally and weekly according to previous studies [74, 75]. This treatment was performed until the final observation week. And for animals treated with FB23-2, 2 mg/mL FB23-2 (*n* = 6) and DMSO vehicle (*N* = 6) were intraperitoneally injected into the mice daily for 4 weeks.

### Statistical analysis

The means and standard deviations of at least three different research were used to calculate the data and error bars. All distinctions between two independent groups were evaluated using a two-tailed Student's t-test. The Kaplan–Meier approach was used to compare the survival curves using the log-rank test. The link between two independent groups was assessed using Pearson's Chi-square. The displayed *P* values (* for *P* < 0.05, ** for *P* < 0.01, *** for *P* < 0.001, and **** for *P* < 0.0001) were determined to be statistically significant.

### Supplementary Information

**Additional file 1: Figure S1. **Expression level and function of FTO in pancreatic cancer.

**Additional file 2: Figure S2. **The inhibitor of FTO (FB23-2) inhibited cell proliferation and increased sensitivity to gemcitabine.

**Additional file 3: Figure S3. **Validation the interaction of USP7 and FTO protein in CFPAC-GM cells.

**Additional file 4: Figure S4. **Validation the interaction of NEDD4 and USP7 protein in CFPAC-GM cells.

**Additional file 5: Figure S5. **Functional analysis of NEDD4 in PDAC cells.

**Additional file 6: Figure S6. **FTO regulated NEDD4 expression in YTHDF2-dependent manner.

**Additional file 7: Figure S7. **Application of FTO inhibitor FB23-2 regulated PI3K/AKT pathway.

**Additional file 8: Figure S8. **Validation of AKT inhibitor in vivo and in vitro.

**Additional file 9:  Figure S9-11.** Quantification of western blot bands.

**Additional file 10.**

**Additional file 11.**

**Additional file 12: Supplementary file 1. **Correlation of FTO and TOP 6 genes in the IP/MS result.

**Additional file 13: Supplementary file 2. **IP result of FTO.

**Additional file 14: Supplementary file 3. **RNA-seq after FTO overexpression in CFPAC-GM cells.

**Additional file 15: Supplementary file 4. **MeRIP-seq of CFPAC-GM cells after FTO overexpression.

**Additional file 16: Supplementary file 5. **RIP-seq with FTO antibody.

**Additional file 17: Supplementary file 6 and 7. **Proteins that potentially interacted with FTO predicted by STRING and Hipredict databases.

**Additional file 18.**

**Additional file 19: Supplementary Table S1. **Sequences of shRNAs used in this study.

**Additional file 20: Supplementary Table S2. **List of primers used in this study.

**Additional file 21: Supplementary Table S3. **List of antibodies.

## Data Availability

The dataset(s) supporting the conclusions of this article is(are) included within the article and its additional files.

## Abbreviations

PDAC: Pancreatic ductal adenocarcinoma
m6A: N6-methyladenosine
CCK8: Cell counting kit-8
IP/MS: Immunoprecipitation/Mass spectrometry
IC50: Inhibitory concentration 50
IHC: Immunohistochemistry
MeRIP-seq: Methylated RNA immunoprecipitation sequencing
TCGA: The cancer genome atlas
CHX: Cycloheximide

## References

[1] Siegel RL, Miller KD, Wagle NS, Jemal A (2023). Cancer statistics, 2023. CA Cancer J Clin.

[2] L.A. Daamen, S.R. de Mol van Otterloo, I. van Goor, H. Eijkelenkamp, B.A. Erickson, W.A. Hall, H.D. Heerkens, G.J. Meijer, I.Q. Molenaar, H.C. van Santvoort, H.M. Verkooijen, M.P.W. Intven, Online adaptive MR-guided stereotactic radiotherapy for unresectable malignancies in the upper abdomen using a 1.5T MR-linac, Acta Oncol 61(1) (2022) 111–115. 10.1080/0284186X.2021.2012593 34879792

[3] Groot VP, Rezaee N, Wu W, Cameron JL, Fishman EK, Hruban RH, Weiss MJ, Zheng L, Wolfgang CL, He J (2018). Patterns, Timing, and Predictors of Recurrence Following Pancreatectomy for Pancreatic Ductal Adenocarcinoma. Ann Surg.

[4] Burris HA, Moore MJ, Andersen J, Green MR, Rothenberg ML, Modiano MR, Cripps MC, Portenoy RK, Storniolo AM, Tarassoff P, Nelson R, Dorr FA, Stephens CD, Von Hoff DD (1997). Improvements in survival and clinical benefit with gemcitabine as first-line therapy for patients with advanced pancreas cancer: a randomized trial. J Clin Oncol.

[5] Heinemann V, Xu YZ, Chubb S, Sen A, Hertel LW, Grindey GB, Plunkett W (1992). Cellular elimination of 2',2'-difluorodeoxycytidine 5'-triphosphate: a mechanism of self-potentiation. Cancer Res.

[6] Hertel LW, Boder GB, Kroin JS, Rinzel SM, Poore GA, Todd GC, Grindey GB (1990). Evaluation of the antitumor activity of gemcitabine (2',2'-difluoro-2'-deoxycytidine). Cancer Res.

[7] Veltkamp SA, Beijnen JH, Schellens JH (2008). Prolonged versus standard gemcitabine infusion: translation of molecular pharmacology to new treatment strategy. Oncologist.

[8] Bergman AM, Pinedo HM, Peters GJ (2002). Determinants of resistance to 2',2'-difluorodeoxycytidine (gemcitabine). Drug Resist Updat.

[9] Manji GA, Olive KP, Saenger YM, Oberstein P (2017). Current and Emerging Therapies in Metastatic Pancreatic Cancer. Clin Cancer Res.

[10] Conroy T, Bachet JB, Ayav A, Huguet F, Lambert A, Caramella C, Maréchal R, Van Laethem JL, Ducreux M (2016). Current standards and new innovative approaches for treatment of pancreatic cancer. Eur J Cancer.

[11] Binenbaum Y, Na'ara S, Gil Z (2015). Gemcitabine resistance in pancreatic ductal adenocarcinoma. Drug Resist Updat.

[12] S. Zeng, M. Pöttler, B. Lan, R. Grützmann, C. Pilarsky, H. Yang, Chemoresistance in Pancreatic Cancer, Int J Mol Sci 20(18) (2019). 10.3390/ijms20184504 PMC6770382 31514451

[13] A. Drakaki, D. Iliopoulos, MicroRNA-gene signaling pathways in pancreatic cancer, Biomedical journal 36(5) (2013). 10.4103/2319-4170.119690 24225187

[14] Neesse A, Michl P, Frese KK, Feig C, Cook N, Jacobetz MA, Lolkema MP, Buchholz M, Olive KP, Gress TM, Tuveson DA (2011). Stromal biology and therapy in pancreatic cancer. Gut.

[15] Kleespies A, Jauch KW, Bruns CJ (2006). Tyrosine kinase inhibitors and gemcitabine: new treatment options in pancreatic cancer?. Drug Resist Updat.

[16] Wiener D, Schwartz S (2021). The epitranscriptome beyond m(6)A. Nat Rev Genet.

[17] Wang T, Kong S, Tao M, Ju S (2020). The potential role of RNA N6-methyladenosine in Cancer progression. Mol Cancer.

[18] Jiang X, Liu B, Nie Z, Duan L, Xiong Q, Jin Z, Yang C, Chen Y (2021). The role of m6A modification in the biological functions and diseases. Signal Transduct Target Ther.

[19] Zhang Y, Chen W, Zheng X, Guo Y, Cao J, Zhang Y, Wen S, Gao W, Wu Y (2021). Regulatory role and mechanism of m(6)A RNA modification in human metabolic diseases. Mol Ther Oncolytics.

[20] Oerum S, Meynier V, Catala M, Tisné C (2021). A comprehensive review of m6A/m6Am RNA methyltransferase structures. Nucleic Acids Res.

[21] Zhao Y, Shi Y, Shen H, Xie W (2020). m(6)A-binding proteins: the emerging crucial performers in epigenetics. J Hematol Oncol.

[22] Zhang Z-W, Zhao X-S, Guo H, Huang X-J (2023). The role of m6A demethylase FTO in chemotherapy resistance mediating acute myeloid leukemia relapse. Cell Death Discovery.

[23] Wang Y, Cheng Z, Xu J, Lai M, Liu L, Zuo M, Dang L (2021). Fat mass and obesity-associated protein (FTO) mediates signal transducer and activator of transcription 3 (STAT3)-drived resistance of breast cancer to doxorubicin. Bioengineered.

[24] Ou B, Liu Y, Gao Z, Xu J, Yan Y, Li Y, Zhang J (2022). Senescent neutrophils-derived exosomal piRNA-17560 promotes chemoresistance and EMT of breast cancer via FTO-mediated m6A demethylation. Cell Death Dis.

[25] Lin Z, Wan AH, Sun L, Liang H, Niu Y, Deng Y, Yan S, Wang QP, Bu X, Zhang X, Hu K, Wan G, He W (2023). N6-methyladenosine demethylase FTO enhances chemo-resistance in colorectal cancer through SIVA1-mediated apoptosis. Mol Ther.

[26] Lan Q, Liu PY, Bell JL, Wang JY, Hüttelmaier S, Zhang XD, Zhang L, Liu T (2021). The Emerging Roles of RNA m(6)A Methylation and Demethylation as Critical Regulators of Tumorigenesis. Drug Sensitivity, and Resistance, Cancer Res.

[27] Wang J, Qiao Y, Sun M, Sun H, Xie F, Chang H, Wang Y, Song J, Lai S, Yang C, Li X, Liu S, Zhao X, Ni K, Meng K, Zhang S, Shan C, Zhang C (2022). FTO promotes colorectal cancer progression and chemotherapy resistance via demethylating G6PD/PARP1. Clin Transl Med.

[28] Zhang S (2018). Mechanism of N(6)-methyladenosine modification and its emerging role in cancer. Pharmacol Ther.

[29] Geng Y, Guan R, Hong W, Huang B, Liu P, Guo X, Hu S, Yu M, Hou B (2020). Identification of m6A-related genes and m6A RNA methylation regulators in pancreatic cancer and their association with survival. Ann Transl Med.

[30] Guo X, Li K, Jiang W, Hu Y, Xiao W, Huang Y, Feng Y, Pan Q, Wan R (2020). RNA demethylase ALKBH5 prevents pancreatic cancer progression by posttranscriptional activation of PER1 in an m6A-YTHDF2-dependent manner. Mol Cancer.

[31] Tang B, Yang Y, Kang M, Wang Y, Wang Y, Bi Y, He S, Shimamoto F (2020). m(6)A demethylase ALKBH5 inhibits pancreatic cancer tumorigenesis by decreasing WIF-1 RNA methylation and mediating Wnt signaling. Mol Cancer.

[32] Xia T, Wu X, Cao M, Zhang P, Shi G, Zhang J, Lu Z, Wu P, Cai B, Miao Y, Jiang K (2019). The RNA m6A methyltransferase METTL3 promotes pancreatic cancer cell proliferation and invasion. Pathol Res Pract.

[33] Huang Y, Su R, Sheng Y, Dong L, Dong Z, Xu H, Ni T, Zhang ZS, Zhang T, Li C, Han L, Zhu Z, Lian F, Wei J, Deng Q, Wang Y, Wunderlich M, Gao Z, Pan G, Zhong D, Zhou H, Zhang N, Gan J, Jiang H, Mulloy JC, Qian Z, Chen J, Yang CG (2019). Small-Molecule Targeting of Oncogenic FTO Demethylase in Acute Myeloid Leukemia. Cancer Cell.

[34] Ali A, Raja R, Farooqui SR, Ahmad S, Banerjea AC (2017). USP7 deubiquitinase controls HIV-1 production by stabilizing Tat protein. Biochem J.

[35] Pozhidaeva A, Bezsonova I (2019). USP7: Structure, substrate specificity, and inhibition. DNA Repair (Amst).

[36] Gu J, Huang W, Wang X, Zhang J, Tao T, Zheng Y, Liu S, Yang J, Chen ZS, Cai CY, Li J, Wang H, Fan Y (2022). Hsa-miR-3178/RhoB/PI3K/Akt, a novel signaling pathway regulates ABC transporters to reverse gemcitabine resistance in pancreatic cancer. Mol Cancer.

[37] K. Okuno, C. Xu, S. Pascual-Sabater, M. Tokunaga, H. Han, C. Fillat, Y. Kinugasa, A. Goel, Berberine Overcomes Gemcitabine-Associated Chemoresistance through Regulation of Rap1/PI3K-Akt Signaling in Pancreatic Ductal Adenocarcinoma, Pharmaceuticals (Basel) 15(10) (2022). 10.3390/ph15101199 PMC9611392 36297310

[38] Zhao F, Yang G, Qiu J, Liu Y, Tao J, Chen G, Su D, You L, Zheng L, Zhang T, Zhao Y (2022). HIF-1α-regulated stanniocalcin-1 mediates gemcitabine resistance in pancreatic ductal adenocarcinoma via PI3K/AKT signaling pathway. Mol Carcinog.

[39] Shi YH, Xu QC, Zhu YQ, Liu ZD, Zhao GY, Liu Q, Wang XY, Wang JQ, Xu X, Su Q, Lai JM, Huang CS, Yin XY (2023). Imatinib facilitates gemcitabine sensitivity by targeting epigenetically activated PDGFC signaling in pancreatic cancer. Mol Ther.

[40] Xing Y, Lin NU, Maurer MA, Chen H, Mahvash A, Sahin A, Akcakanat A, Li Y, Abramson V, Litton J, Chavez-MacGregor M, Valero V, Piha-Paul SA, Hong D, Do KA, Tarco E, Riall D, Eterovic AK, Wulf GM, Cantley LC, Mills GB, Doyle LA, Winer E, Hortobagyi GN, Gonzalez-Angulo AM, Meric-Bernstam F (2019). Phase II trial of AKT inhibitor MK-2206 in patients with advanced breast cancer who have tumors with PIK3CA or AKT mutations, and/or PTEN loss/PTEN mutation. Breast Cancer Res.

[41] M. Chen, Y. Yu, T. Mi, Q. Guo, B. Xiang, X. Tian, L. Jin, C. Long, L. Shen, X. Liu, J. Pan, Y. Zhang, T. Xu, D. Zhang, G. Wei, MK-2206 Alleviates Renal Fibrosis by Suppressing the Akt/mTOR Signaling Pathway In Vivo and In Vitro, Cells 11(21) (2022). 10.3390/cells11213505 PMC9655032 36359901

[42] Wang Z, Luo G, Qiu Z (2020). Akt inhibitor MK-2206 reduces pancreatic cancer cell viability and increases the efficacy of gemcitabine. Oncol Lett.

[43] Bjune K, Sundvold H, Leren TP, Naderi S (2018). MK-2206, an allosteric inhibitor of AKT, stimulates LDLR expression and LDL uptake: A potential hypocholesterolemic agent. Atherosclerosis.

[44] Masuya D, Huang C, Liu D, Nakashima T, Yokomise H, Ueno M, Nakashima N, Sumitomo S (2006). The HAUSP gene plays an important role in non-small cell lung carcinogenesis through p53-dependent pathways. J Pathol.

[45] Bhattacharya S, Chakraborty D, Basu M, Ghosh MK (2018). Emerging insights into HAUSP (USP7) in physiology, cancer and other diseases. Signal Transduct Target Ther.

[46] Hernández-Pérez S, Cabrera E, Salido E, Lim M, Reid L, Lakhani SR, Khanna KK, Saunus JM, Freire R (2017). DUB3 and USP7 de-ubiquitinating enzymes control replication inhibitor Geminin: molecular characterization and associations with breast cancer. Oncogene.

[47] S. Sundara Rajan, A.M. Hanby, K. Horgan, H.H. Thygesen, V. Speirs, The potential utility of geminin as a predictive biomarker in breast cancer, Breast Cancer Res Treat 143(1) (2014) 91–8. 10.1007/s10549-013-2786-5 24292956

[48] Chen H, Zhu X, Sun R, Ma P, Zhang E, Wang Z, Fan Y, Zhou G, Mao R (2020). Ubiquitin-specific protease 7 is a druggable target that is essential for pancreatic cancer growth and chemoresistance. Invest New Drugs.

[49] Jia G, Fu Y, Zhao X, Dai Q, Zheng G, Yang Y, Yi C, Lindahl T, Pan T, Yang YG, He C (2011). N6-methyladenosine in nuclear RNA is a major substrate of the obesity-associated FTO. Nat Chem Biol.

[50] Church C, Moir L, McMurray F, Girard C, Banks GT, Teboul L, Wells S, Brüning JC, Nolan PM, Ashcroft FM, Cox RD (2010). Overexpression of Fto leads to increased food intake and results in obesity. Nat Genet.

[51] Shen F, Huang W, Huang JT, Xiong J, Yang Y, Wu K, Jia GF, Chen J, Feng YQ, Yuan BF, Liu SM (2015). Decreased N(6)-methyladenosine in peripheral blood RNA from diabetic patients is associated with FTO expression rather than ALKBH5. J Clin Endocrinol Metab.

[52] Mathiyalagan P, Adamiak M, Mayourian J, Sassi Y, Liang Y, Agarwal N, Jha D, Zhang S, Kohlbrenner E, Chepurko E, Chen J, Trivieri MG, Singh R, Bouchareb R, Fish K, Ishikawa K, Lebeche D, Hajjar RJ, Sahoo S (2019). FTO-Dependent N(6)-Methyladenosine Regulates Cardiac Function During Remodeling and Repair. Circulation.

[53] Han M, Liu Z, Xu Y, Liu X, Wang D, Li F, Wang Y, Bi J (2020). Abnormality of m6A mRNA Methylation Is Involved in Alzheimer's Disease. Front Neurosci.

[54] Hess ME, Hess S, Meyer KD, Verhagen LA, Koch L, Brönneke HS, Dietrich MO, Jordan SD, Saletore Y, Elemento O, Belgardt BF, Franz T, Horvath TL, Rüther U, Jaffrey SR, Kloppenburg P, Brüning JC (2013). The fat mass and obesity associated gene (Fto) regulates activity of the dopaminergic midbrain circuitry. Nat Neurosci.

[55] Tan Z, Shi S, Xu J, Liu X, Lei Y, Zhang B, Hua J, Meng Q, Wang W, Yu X, Liang C (2022). RNA N6-methyladenosine demethylase FTO promotes pancreatic cancer progression by inducing the autocrine activity of PDGFC in an m(6)A-YTHDF2-dependent manner. Oncogene.

[56] Feng S, Yang G, Yang H, Liang Z, Zhang R, Fan Y, Zhang G (2017). NEDD4 is involved in acquisition of epithelial-mesenchymal transition in cisplatin-resistant nasopharyngeal carcinoma cells. Cell Cycle.

[57] Sun H, Ma H, Wang J, Xia L, Zhu G, Wang Z, Sun J, Chen Z (2017). Phosphatase and tensin homolog deleted on chromosome 10 degradation induced by NEDD4 promotes acquired erlotinib resistance in non-small-cell lung cancer. Tumour Biol.

[58] Thummuri D, Khan S, Underwood PW, Zhang P, Wiegand J, Zhang X, Budamagunta V, Sobh A, Tagmount A, Loguinov A, Riner AN, Akki AS, Williamson E, Hromas R, Vulpe CD, Zheng G, Trevino JG, Zhou D (2022). Overcoming Gemcitabine Resistance in Pancreatic Cancer Using the BCL-X(L)-Specific Degrader DT2216. Mol Cancer Ther.

[59] Sarvepalli D, Rashid MU, Rahman AU, Ullah W, Hussain I, Hasan B, Jehanzeb S, Khan AK, Jain AG, Khetpal N, Ahmad S (2019). Gemcitabine: A Review of Chemoresistance in Pancreatic Cancer. Crit Rev Oncog.

[60] Fruman DA, Chiu H, Hopkins BD, Bagrodia S, Cantley LC, Abraham RT (2017). The PI3K Pathway in Human Disease. Cell.

[61] Gulluni F, De Santis MC, Margaria JP, Martini M, Hirsch E (2019). Class II PI3K Functions in Cell Biology and Disease. Trends Cell Biol.

[62] Gozzelino L, De Santis MC, Gulluni F, Hirsch E, Martini M (2020). PI(3,4)P2 Signaling in Cancer and Metabolism. Front Oncol.

[63] Kennedy AL, Morton JP, Manoharan I, Nelson DM, Jamieson NB, Pawlikowski JS, McBryan T, Doyle B, McKay C, Oien KA, Enders GH, Zhang R, Sansom OJ, Adams PD (2011). Activation of the PIK3CA/AKT pathway suppresses senescence induced by an activated RAS oncogene to promote tumorigenesis. Mol Cell.

[64] Thibault B, Ramos-Delgado F, Pons-Tostivint E, Therville N, Cintas C, Arcucci S, Cassant-Sourdy S, Reyes-Castellanos G, Tosolini M, Villard AV, Cayron C, Baer R, Bertrand-Michel J, Pagan D, Ferreira Da Mota D, Yan H, Falcomatà C, Muscari F, Bournet B, Delord JP, Aksoy E, Carrier A, Cordelier P, Saur D, Basset C, Guillermet-Guibert J (2021). Pancreatic cancer intrinsic PI3Kα activity accelerates metastasis and rewires macrophage component. EMBO Mol Med.

[65] Manning BD, Cantley LC (2007). AKT/PKB signaling: navigating downstream. Cell.

[66] Larribere L, Khaled M, Tartare-Deckert S, Busca R, Luciano F, Bille K, Valony G, Eychene A, Auberger P, Ortonne JP, Ballotti R, Bertolotto C (2004). PI3K mediates protection against TRAIL-induced apoptosis in primary human melanocytes. Cell Death Differ.

[67] Li YC, He SM, He ZX, Li M, Yang Y, Pang JX, Zhang X, Chow K, Zhou Q, Duan W, Zhou ZW, Yang T, Huang GH, Liu A, Qiu JX, Liu JP, Zhou SF (2014). Plumbagin induces apoptotic and autophagic cell death through inhibition of the PI3K/Akt/mTOR pathway in human non-small cell lung cancer cells. Cancer Lett.

[68] Zhao M, Luo R, Liu Y, Gao L, Fu Z, Fu Q, Luo X, Chen Y, Deng X, Liang Z, Li X, Cheng C, Liu Z, Fang W (2016). miR-3188 regulates nasopharyngeal carcinoma proliferation and chemosensitivity through a FOXO1-modulated positive feedback loop with mTOR-p-PI3K/AKT-c-JUN. Nat Commun.

[69] Park MK, Yao Y, Xia W, Setijono SR, Kim JH, Vila IK, Chiu HH, Wu Y, Billalabeitia EG, Lee MG, Kalb RG, Hung MC, Pandolfi PP, Song SJ, Song MS (2019). PTEN self-regulates through USP11 via the PI3K-FOXO pathway to stabilize tumor suppression. Nat Commun.

[70] Liang S, Guo H, Ma K, Li X, Wu D, Wang Y, Wang W, Zhang S, Cui Y, Liu Y, Sun L, Zhang B, Xin M, Zhang N, Zhou H, Liu Y, Wang J, Liu L (2021). A PLCB1-PI3K-AKT Signaling Axis Activates EMT to Promote Cholangiocarcinoma Progression. Cancer Res.

[71] Xu F, Huang M, Chen Q, Niu Y, Hu Y, Hu P, Chen D, He C, Huang K, Zeng Z, Tang J, Wang F, Zhao Y, Wang C, Zhao G (2021). LncRNA HIF1A-AS1 Promotes Gemcitabine Resistance of Pancreatic Cancer by Enhancing Glycolysis through Modulating the AKT/YB1/HIF1α Pathway. Cancer Res.

[72] Morgan RT, Woods LK, Moore GE, Quinn LA, McGavran L, Gordon SG (1980). Human cell line (COLO 357) of metastatic pancreatic adenocarcinoma. Int J Cancer.

[73] Dominissini D, Moshitch-Moshkovitz S, Salmon-Divon M, Amariglio N, Rechavi G (2013). Transcriptome-wide mapping of N(6)-methyladenosine by m(6)A-seq based on immunocapturing and massively parallel sequencing. Nat Protoc.

[74] Higuchi T, Yokobori T, Naito T, Kakinuma C, Hagiwara S, Nishiyama M, Asao T (2018). Investigation into metastatic processes and the therapeutic effects of gemcitabine on human pancreatic cancer using an orthotopic SUIT-2 pancreatic cancer mouse model. Oncol Lett.

[75] Veerman G, Ruiz van Haperen VW, Vermorken JB, Noordhuis P, Braakhuis BJ, Pinedo HM, Peters GJ (1996). Antitumor activity of prolonged as compared with bolus administration of 2',2'-difluorodeoxycytidine in vivo against murine colon tumors. Cancer Chemother Pharmacol.

