# Supplementary figures and images for: m6A eraser FTO impairs gemcitabine resistance in pancreatic cancer through influencing NEDD4 mRNA stability by regulating the PTEN/PI3K/AKT pathway
Source: J Exp Clin Cancer Res. 2023 Aug 22;42:217. doi: 10.1186/s13046-023-02792-0 (PMC10464189; doi:10.1186/s13046-023-02792-0)

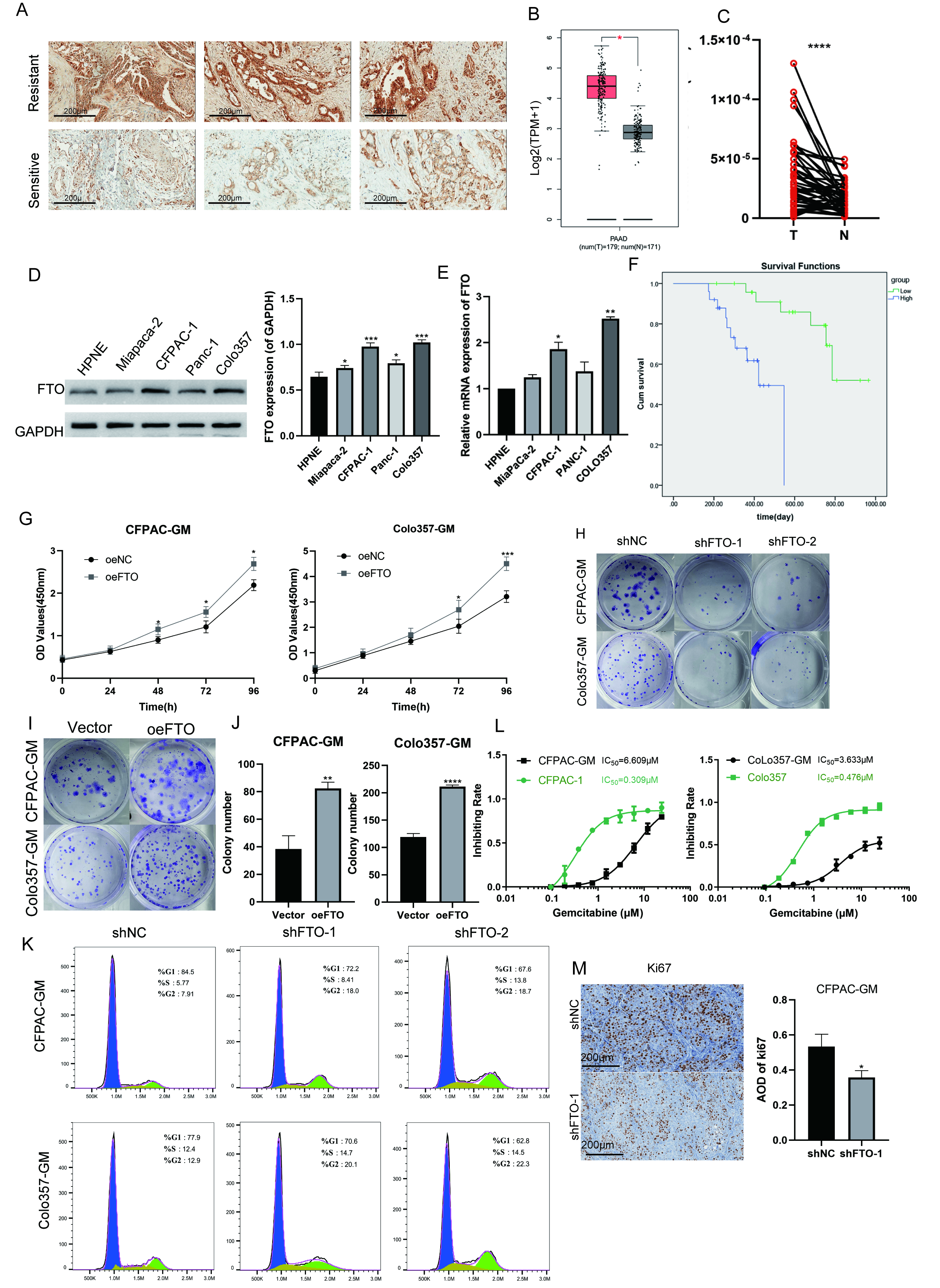

Supplement: Supplementary file 1 — Additional file 1: Figure S1. Expression level and function of FTO in pancreatic cancer. [file 13046_2023_2792_MOESM1_ESM.tif]

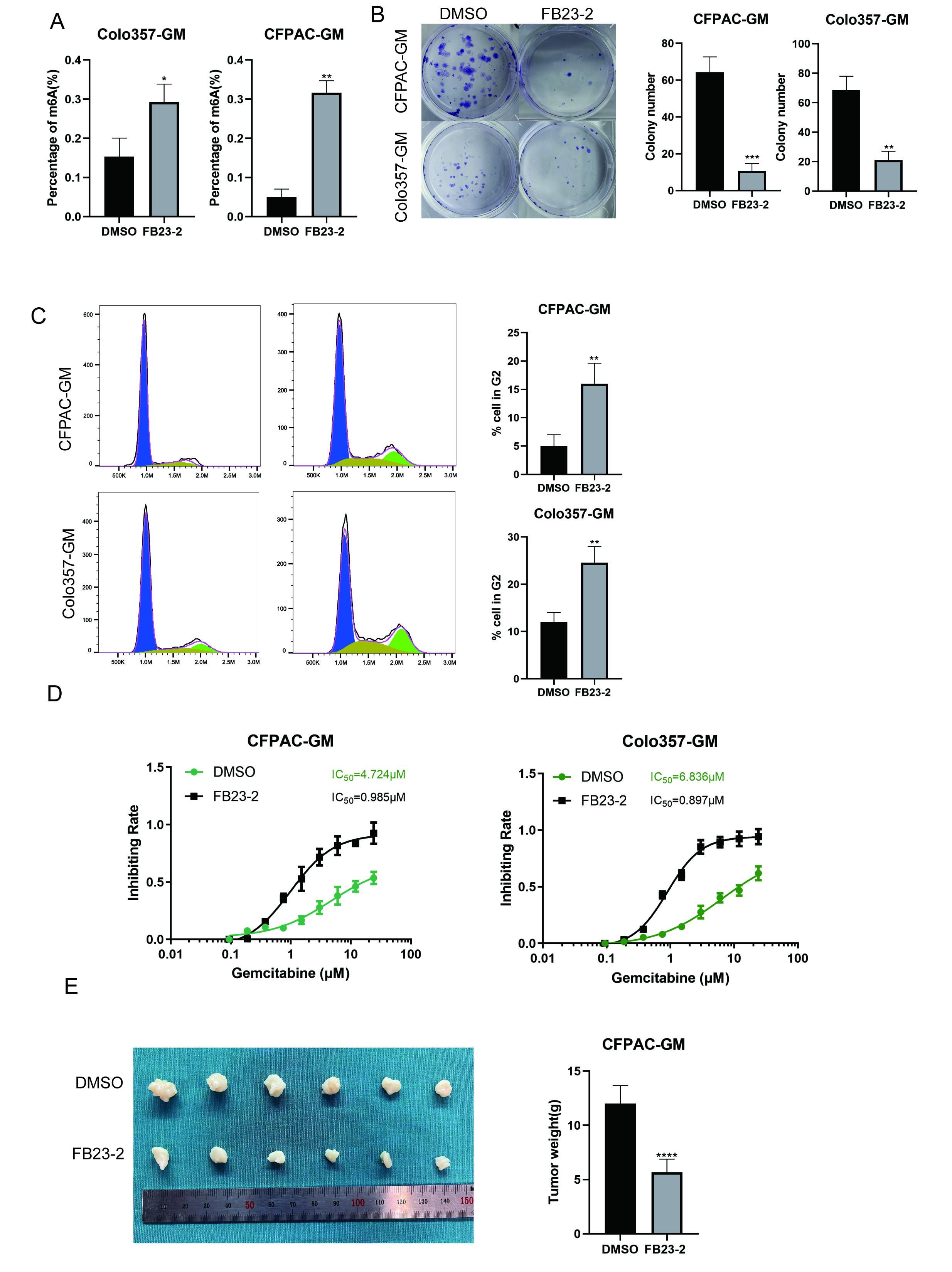

Supplement: Supplementary file 2 — Additional file 2: Figure S2. The inhibitor of FTO (FB23-2) inhibited cell proliferation and increased sensitivity to gemcitabine. [file 13046_2023_2792_MOESM2_ESM.tif]

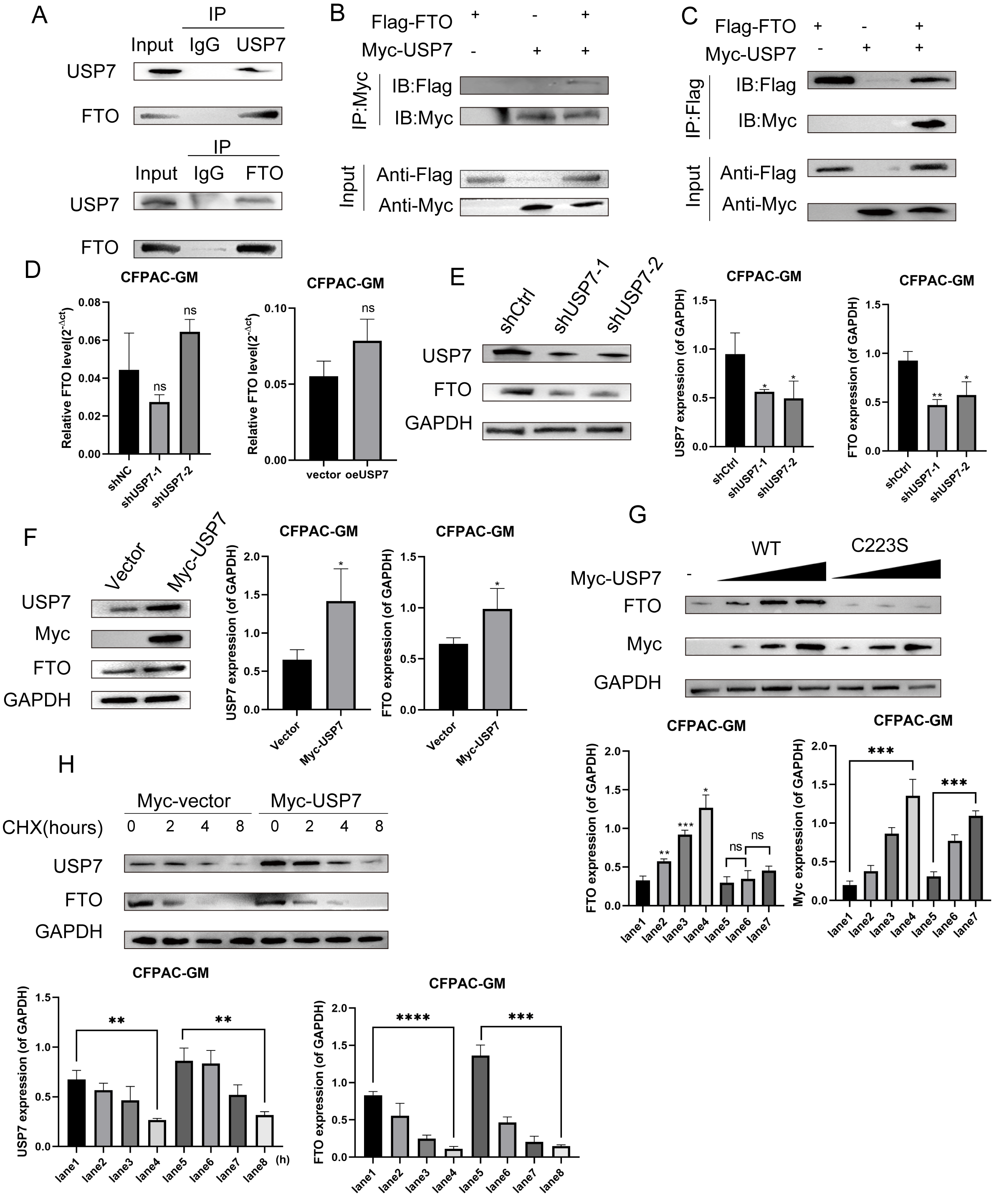

Supplement: Supplementary file 3 — Additional file 3: Figure S3. Validation the interaction of USP7 and FTO protein in CFPAC-GM cells. [file 13046_2023_2792_MOESM3_ESM.tif]

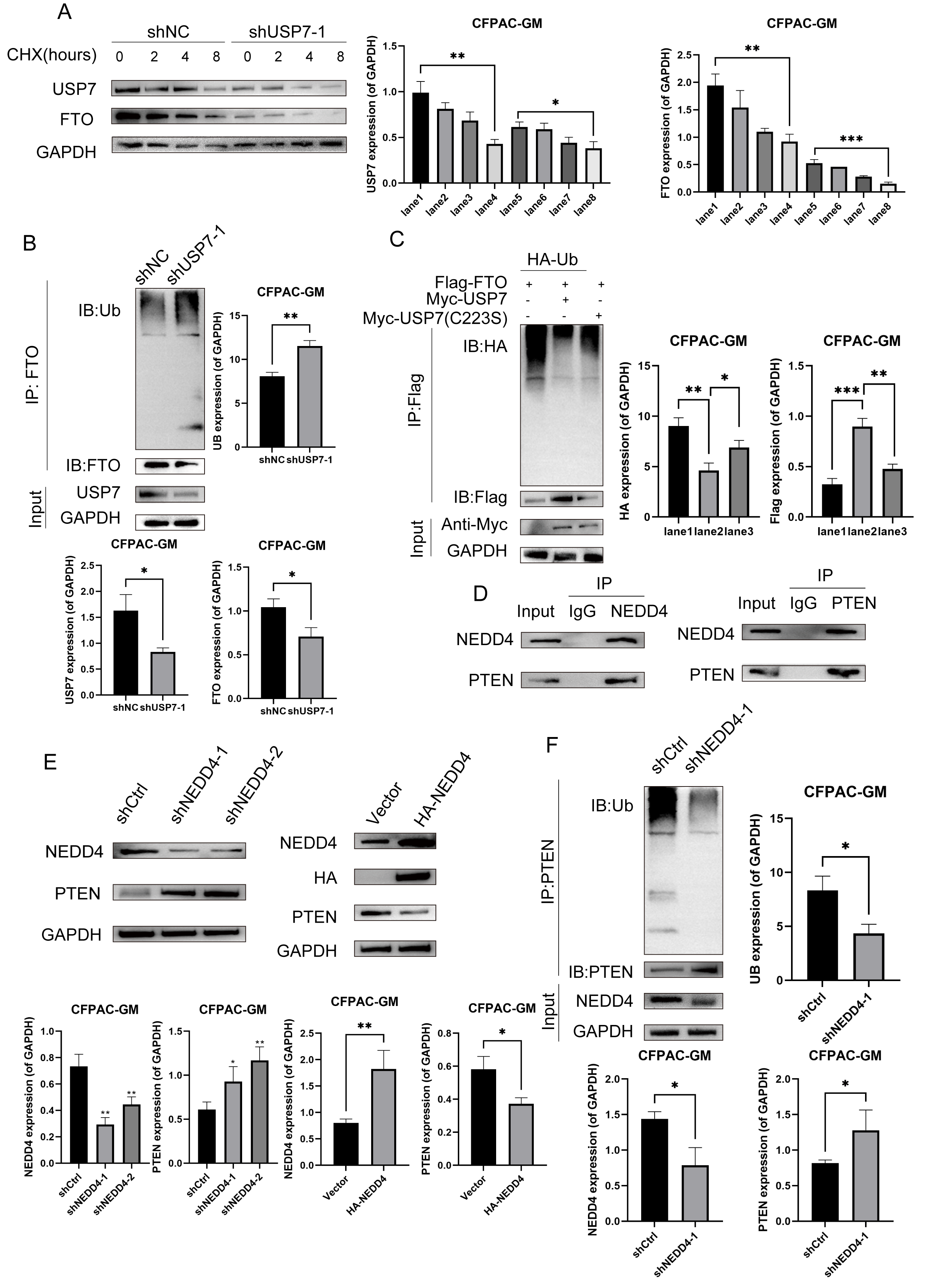

Supplement: Supplementary file 4 — Additional file 4: Figure S4. Validation the interaction of NEDD4 and USP7 protein in CFPAC-GM cells. [file 13046_2023_2792_MOESM4_ESM.tif]

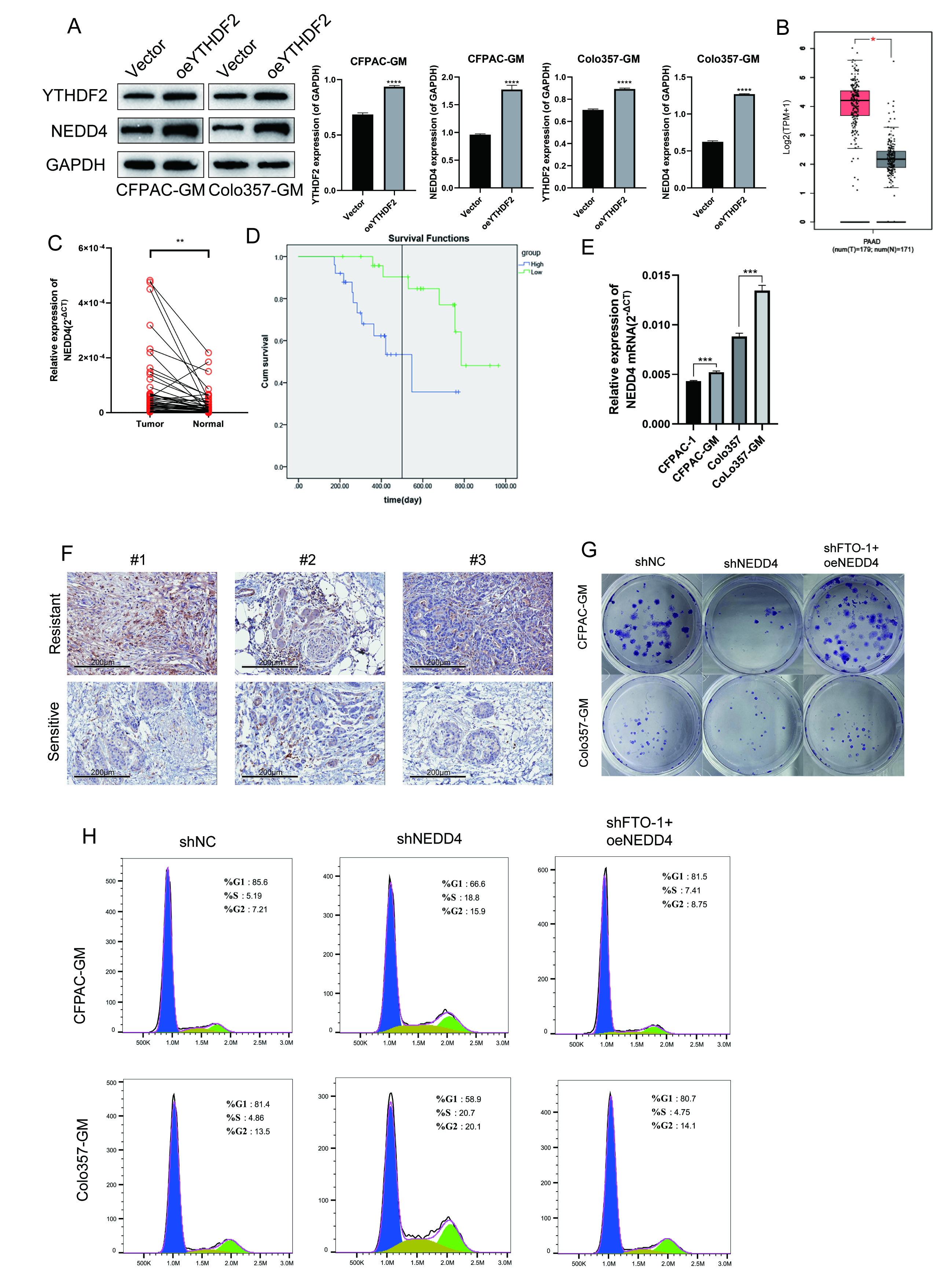

Supplement: Supplementary file 5 — Additional file 5: Figure S5. Functional analysis of NEDD4 in PDAC cells. [file 13046_2023_2792_MOESM5_ESM.tif]

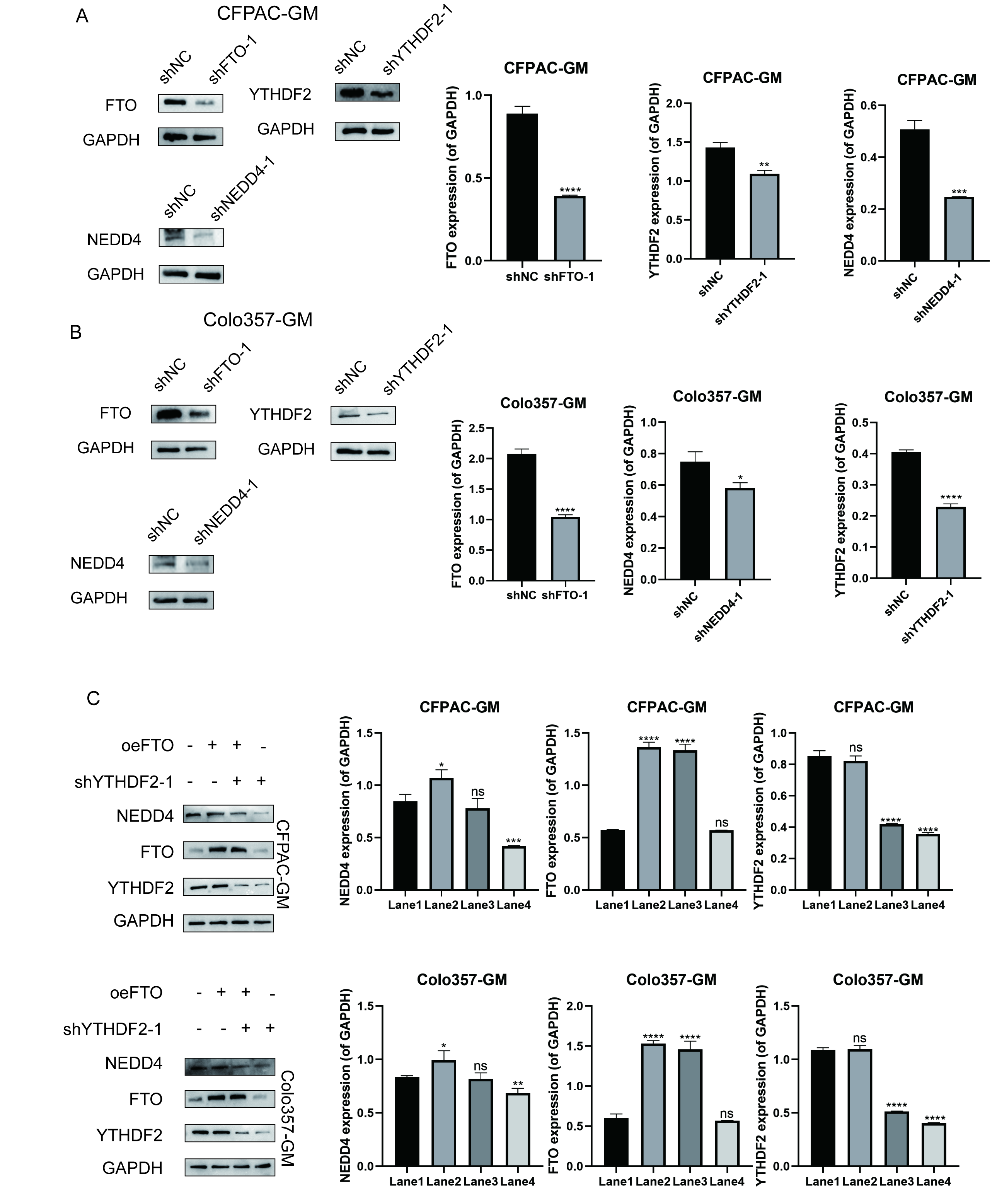

Supplement: Supplementary file 6 — Additional file 6: Figure S6. FTO regulated NEDD4 expression in YTHDF2-dependent manner. [file 13046_2023_2792_MOESM6_ESM.tif]

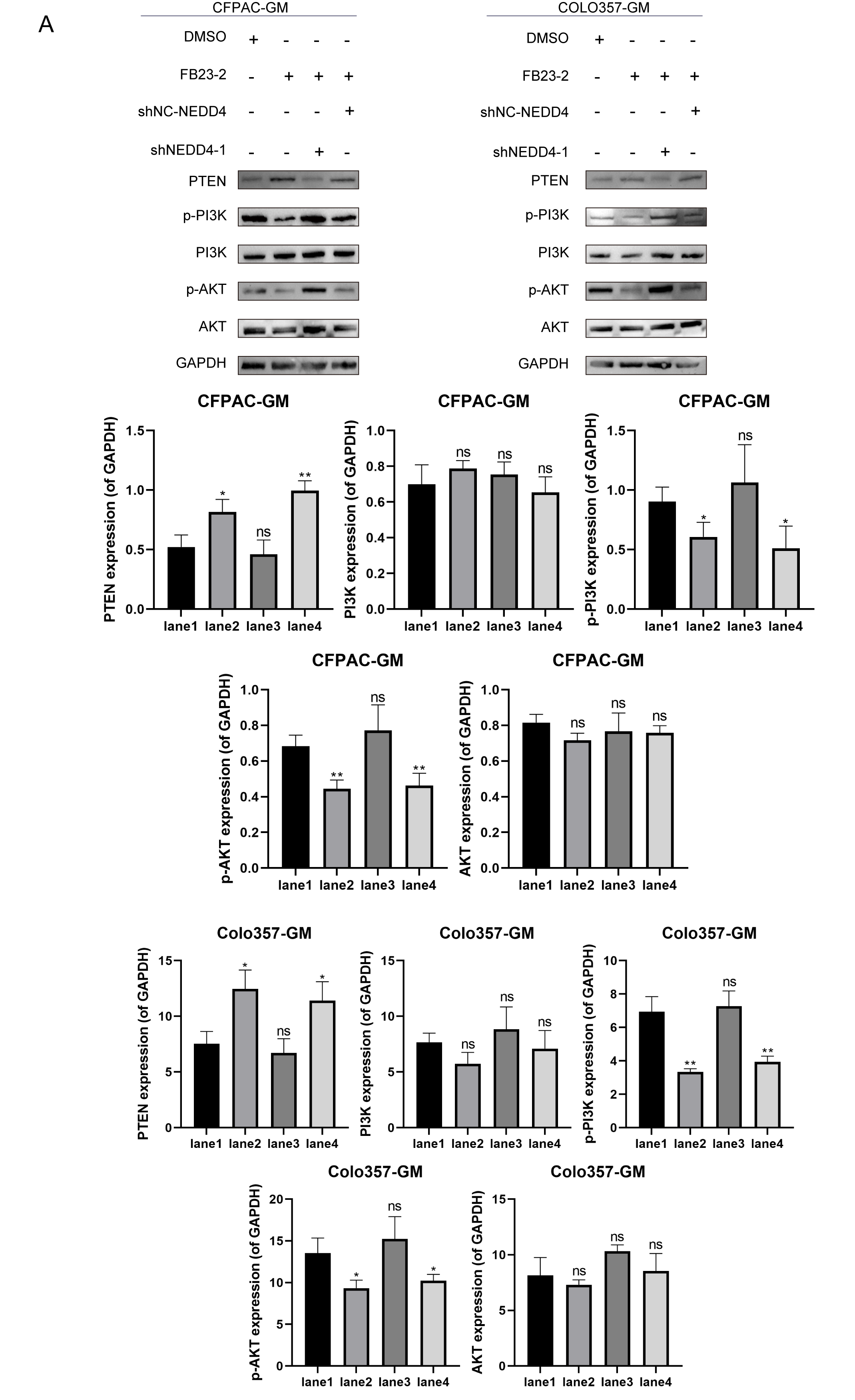

Supplement: Supplementary file 7 — Additional file 7: Figure S7. Application of FTO inhibitor FB23-2 regulated PI3K/AKT pathway. [file 13046_2023_2792_MOESM7_ESM.tif]

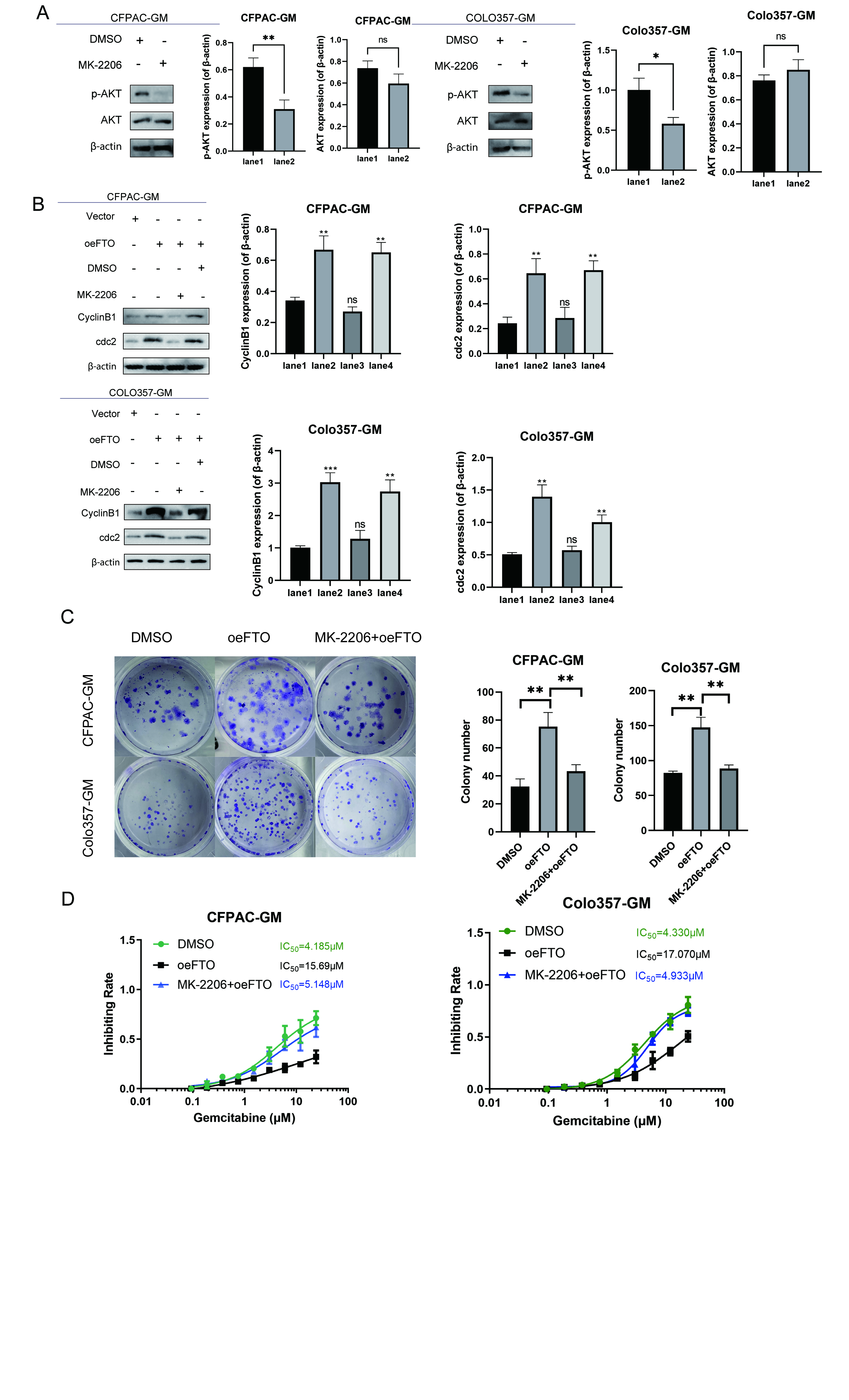

Supplement: Supplementary file 8 — Additional file 8: Figure S8. Validation of AKT inhibitor in vivo and in vitro. [file 13046_2023_2792_MOESM8_ESM.tif]

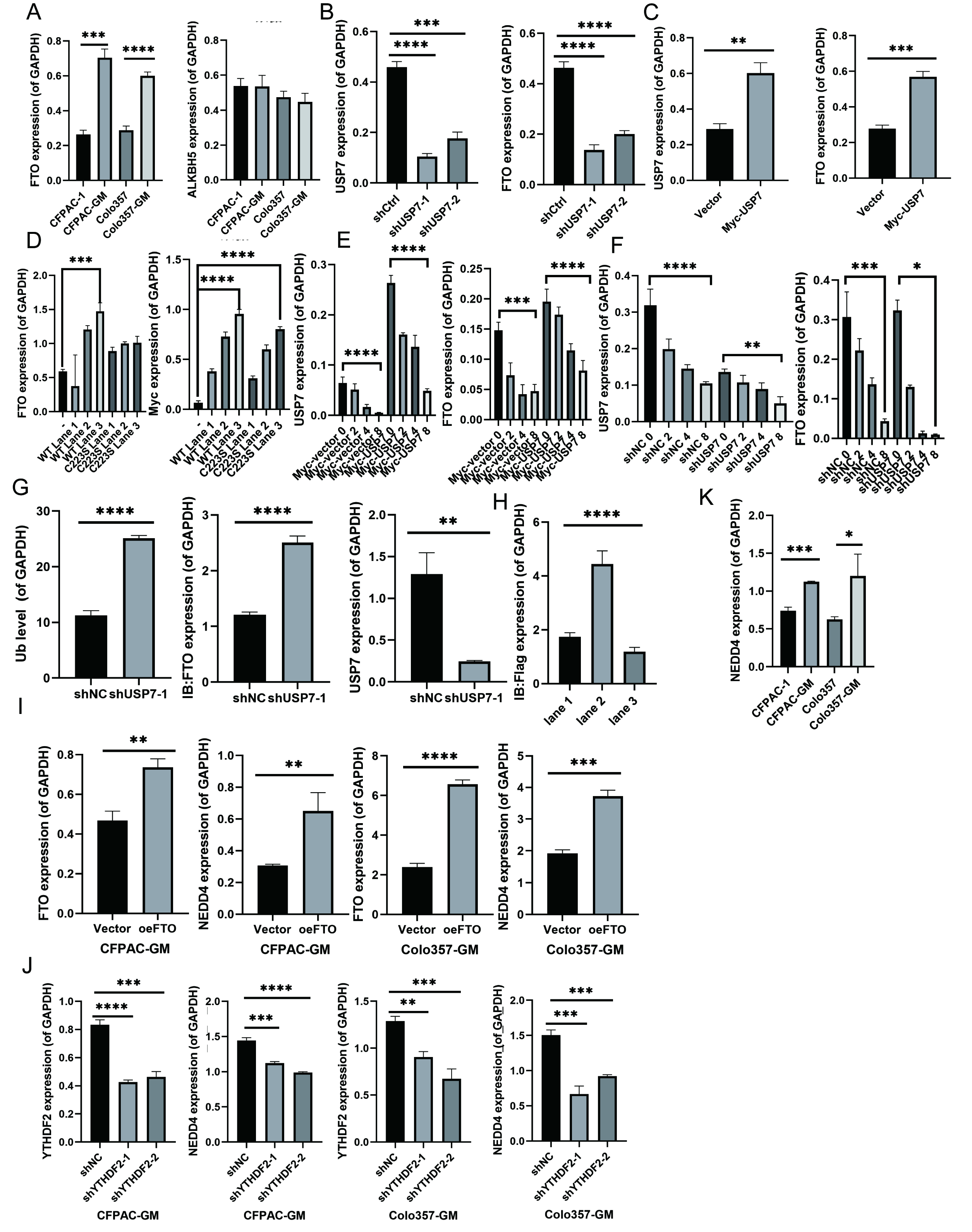

Supplement: Supplementary file 9 — Additional file 9: Figure S9-11. Quantification of western blot bands. [file 13046_2023_2792_MOESM9_ESM.tif]

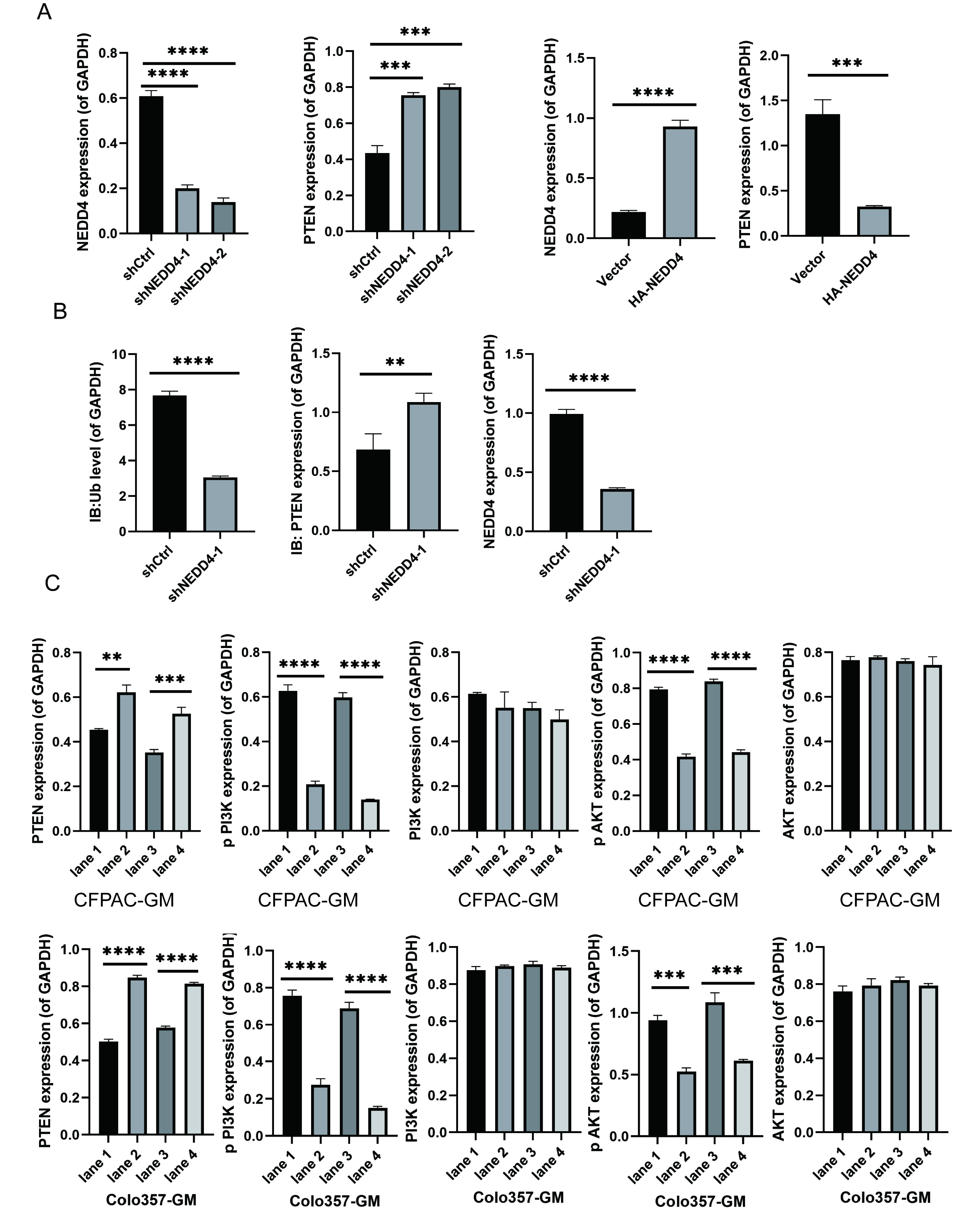

Supplement: Supplementary file 10 — Additional file 10. [file 13046_2023_2792_MOESM10_ESM.tif]

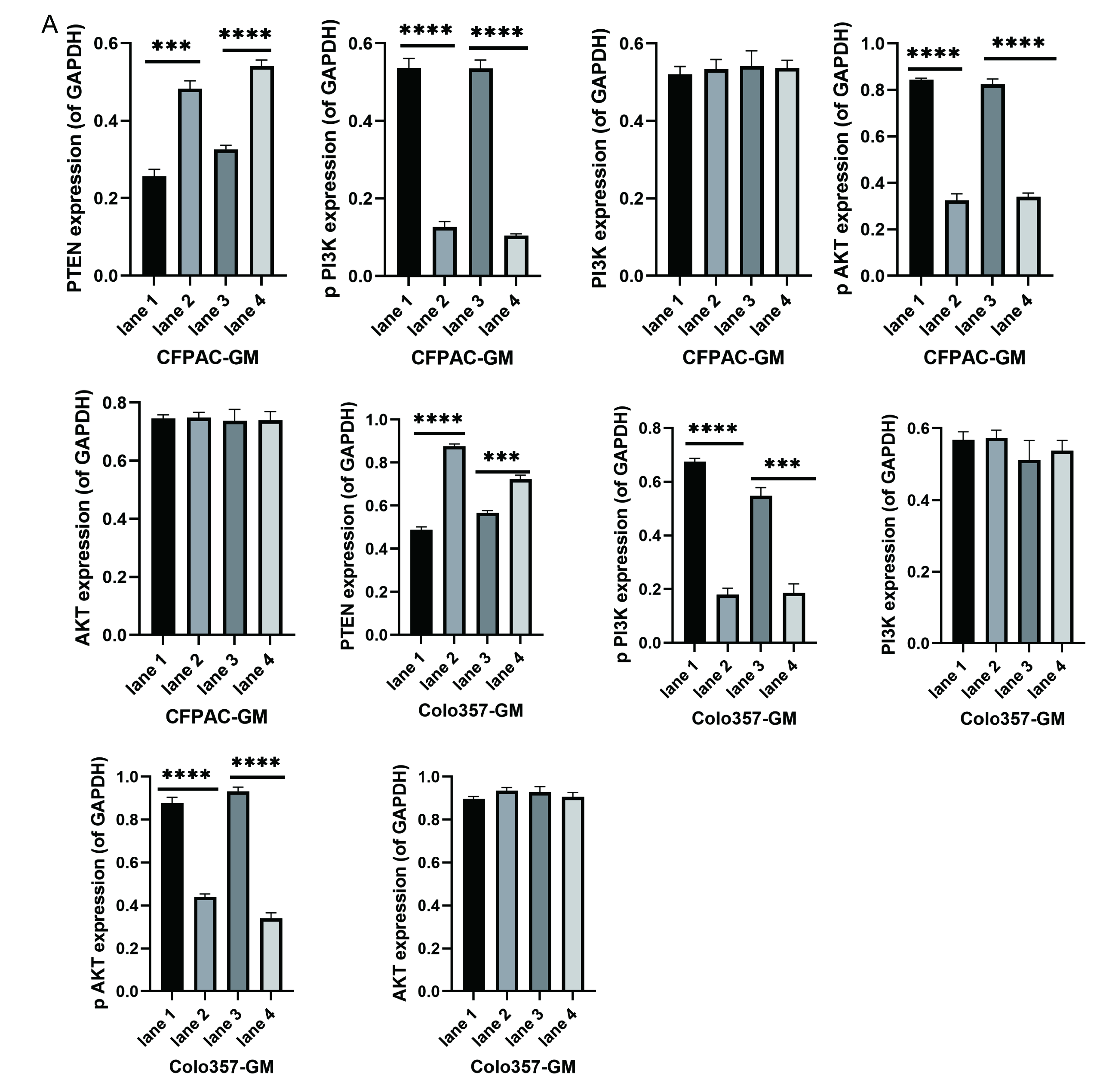

Supplement: Supplementary file 11 — Additional file 11. [file 13046_2023_2792_MOESM11_ESM.tif]

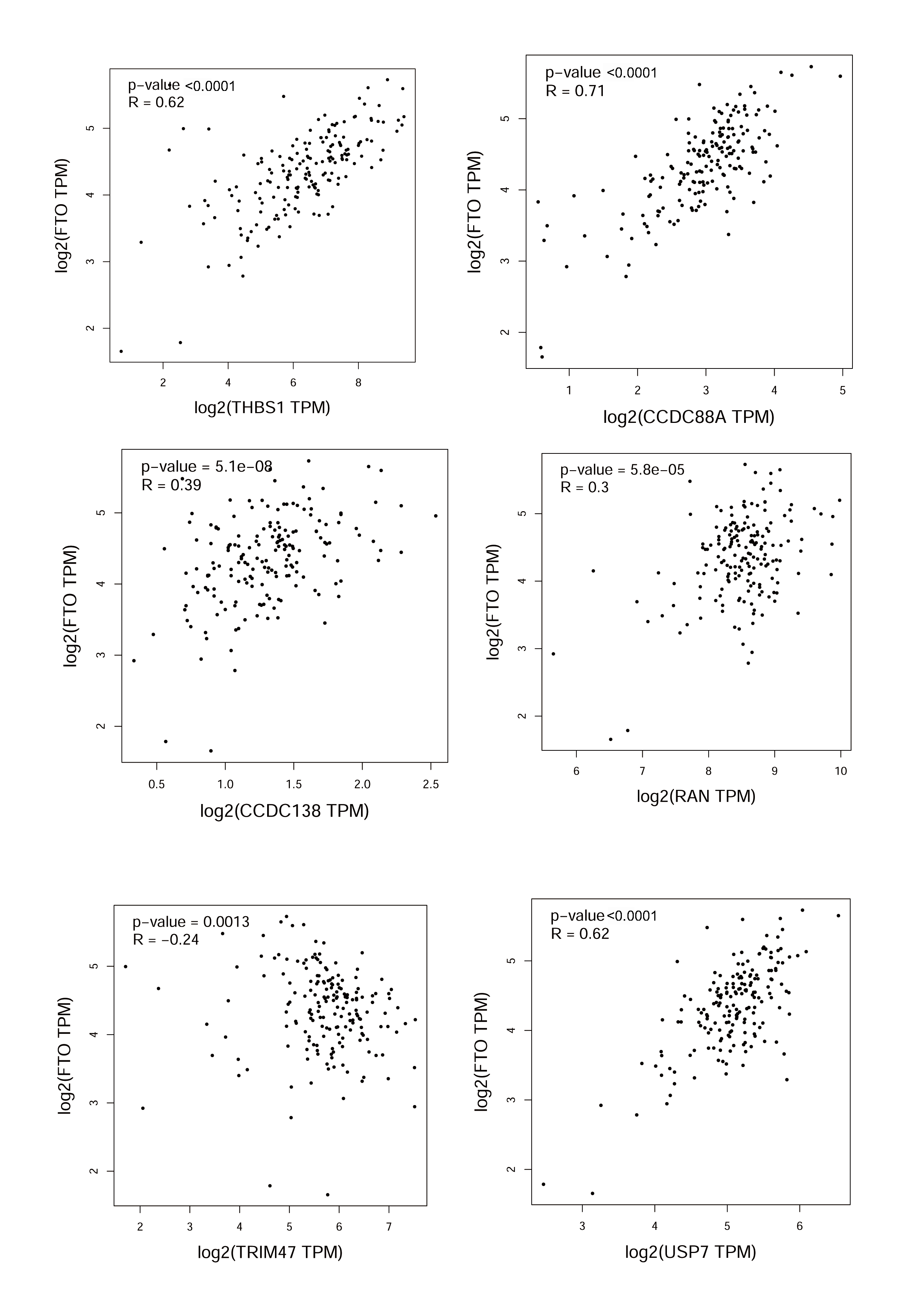

Supplement: Supplementary file 12 — Additional file 12: Supplementary file 1. Correlation of FTO and TOP 6 genes in the IP/MS result. [file 13046_2023_2792_MOESM12_ESM.tif]
